# Phenotypic heterogeneity and plasticity in colorectal cancer metastasis

**DOI:** 10.1016/j.xgen.2025.100881

**Published:** 2025-05-19

**Authors:** Samuel Ogden, Nasrine Metic, Ozen Leylek, Elise A. Smith, Alison M. Berner, Ann-Marie Baker, Imran Uddin, Marta Buzzetti, Marco Gerlinger, Cancer Tissue Bank, Trevor Graham, Hemant M. Kocher, Mirjana Efremova

**Affiliations:** 1 Barts Cancer Institute, Queen Mary University of London, London, UK; 2 The Institute of Cancer Research, London, UK; 3 CRUK City of London Centre Single Cell Genomics Facility, University College London, London, UK

**Keywords:** phenotypic heterogeneity, colorectal cancer, metastasis, plasticity, single-cell multiomics, AP-1, NOTUM, spatial transcriptomics

## Abstract

Phenotypic heterogeneity and plasticity in colorectal cancer (CRC) has a crucial role in tumor progression, metastasis, and therapy resistance. However, the regulatory factors and the extrinsic signals driving phenotypic heterogeneity remain unknown. Using a combination of single-cell multiomics and spatial transcriptomics data from primary and metastatic CRC patients, we reveal cancer cell states with regenerative and inflammatory phenotypes that closely resemble metastasis-initiating cells in mouse models. We identify an intermediate population with a hybrid regenerative and stem phenotype. We reveal the transcription factors AP-1 and nuclear factor κB (NF-κB) as their key regulators and show localization of these states in an immunosuppressive niche both at the invasive edge in primary CRC and in liver metastasis. We uncover ligand-receptor interactions predicted to activate the regenerative and inflammatory phenotype in cancer cells. Together, our findings reveal regulatory and signaling factors that mediate distinct cancer cell states and can serve as potential targets to impair metastasis.

## Highlights

• Single-cell multiomics and spatial data are generated from metastatic colorectal samples
• Regenerative and inflammatory cancer cell states are revealed
• AP-1 and NF-κB are identified as key regulators of the regenerative cell states
• Regenerative cells are localized at the invasive edge in an immunosuppressive niche

## Graphical abstract

**Figure:**
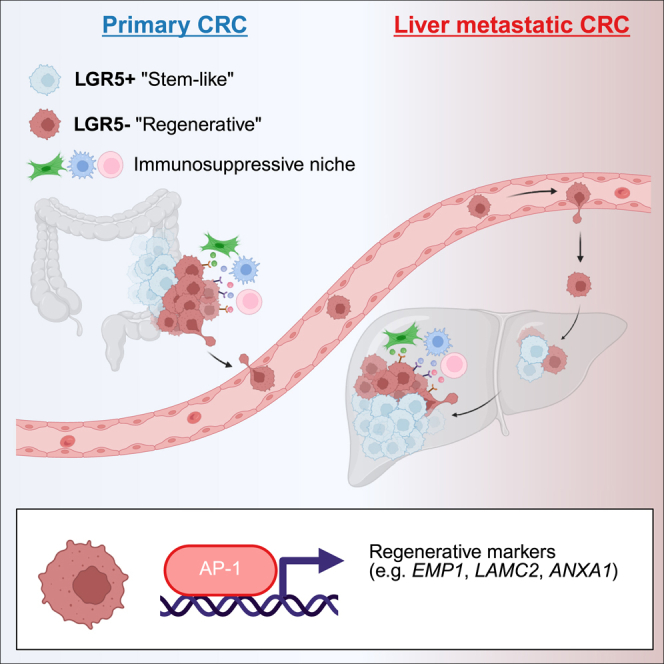

## Teaser

Ogden and colleagues reveal regenerative and inflammatory cancer cell states in primary and metastatic colorectal cancer. They combine single-cell multiomics and spatial transcriptomics patient data to identify key transcription factors and signals from the tumor microenvironment that drive these cancer cell states.

## Introduction

Colorectal cancer is the third most common malignancy globally and the second leading cause of cancer-related death. Phenotypic plasticity—the ability of cells to undergo rapid phenotypic transitions in response to external signals and adapt to new microenvironments—plays a key role in colorectal cancer (CRC) progression and therapy resistance.^1^^,^^2^

CRC has a cellular hierarchy resembling a healthy intestine,^3^ maintained by LGR5-expressing stem cells that give rise to transient-amplifying (TA) progenitors that undergo differentiation into absorptive and secretory lineages. However, following tissue damage or loss of stem cells, differentiated cells can dedifferentiate and replenish the impaired stem cell niches to enable tissue repair.^4^ The same phenomenon has been described in murine CRC models after ablation of Lgr5^+^ stem cells.^5^ A regenerative stem cell (RSC) or revival stem cell state (revSCS) has been shown to be central to this process of repair^6^^,^^7^^,^^8^ and has been identified both in primary CRC (pCRC) mouse models and patient samples.^9^^,^^10^^,^^11^ Furthermore, mouse model studies have shown that disseminating cells are predominantly Lgr5^−^ regenerative-like cells that restore the Lgr5^+^ stem cell phenotype at metastatic sites to progress into macrometastasis.^12^^,^^13^^,^^14^ Emerging data from human metastatic samples show progressive metastatic plasticity that enables differentiation into non-canonical squamous and neuroendocrine-like states and that this process is enhanced by chemotherapy.^15^ Such reversible cell state transitions indicate that cellular reprogramming is largely driven by epigenetic plasticity that can initiate new transcriptional programs in response to external signals.^16^ However, the regulatory factors and extrinsic signals driving this heterogeneity are poorly understood. This hinders efforts to improve prognostication, predict who will benefit from treatment, and develop new therapies.

Here, we sought to characterize the heterogeneous cancer cell states in primary and liver metastatic CRC and investigate the intrinsic and extrinsic factors that drive those states, using a combination of single-cell RNA-seq (scRNA-seq), single-nucleus (sn) multiomics, and spatial transcriptomics data. We find diverse subsets of regenerative cells with an inflammatory or hybrid stem-regenerative phenotype and identify transcription factors driving those states. We show that these inflammatory regenerative states are enriched at the tumor invasive front and surrounded by an immunosuppressive niche. We further uncover ligand-receptor interactions, driven by cancer-associated fibroblasts (CAFs), macrophages, and CD8 T cells, potentially activating and sustaining this program.

## Results

### Regenerative inflammatory cancer cell states in primary CRC

To examine the cellular heterogeneity of malignant cell states and the tumor microenvironment (TME) in pCRC and see how this landscape changes in liver metastasis (LM), we integrated published pCRC scRNA-seq data^17^^,^^18^^,^^19^ and generated LM single-nucleus Multiome RNA + assay for transposase-accessible chromatin (ATAC) data.

**Figure 1:**
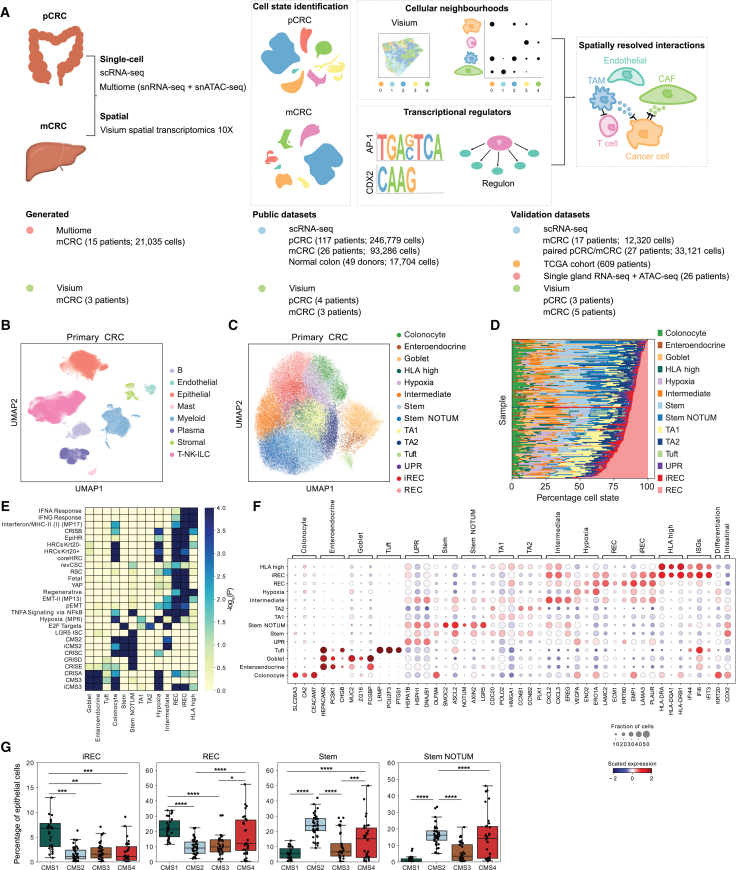
Heterogeneous cancer cell states in pCRC (A) Experimental design. (B) UMAP representation showing cell types in pCRC datasets.^17^^,^^18^^,^^19^^,^^20^ (C) UMAP representation showing malignant pCRC states. (D) Proportions of cancer states in pCRC. (E) GEA of differentially expressed genes (DEGs) in cancer states for the indicated signatures: IFN/major histocompatibility complex (MHC) class II, hypoxia, and EMT-II^101^; CRIS^21^; EpiHR and coreHRC^14^; revCSC^10^; RSC^9^; fetal^112^; YAP^113^; regenerative^8^; pEMT^114^; CMS2 and CMS3^115^; iCMS2 and iCMS3^25^; and MSigDB Hallmarks (Table S2). (F) Scaled mRNA expression of marker genes in pCRC. (G) The percentage of cancer states in the epithelial compartment for each CMS. Unpaired t test: ∗*p* < 0.05,∗∗*p* < 0.01, ∗∗∗*p* < 0.001, ∗∗∗∗*p* < 0.0001.

Focusing first on pCRC, we integrated data from 117 untreated CRC patients,^17^^,^^18^^,^^20^ and after quality control, we retained transcriptomes from 246,779 cells, including cancer, immune, and stromal cells (Figures 1A, 1B, and S1A–S1C). Subclustering of malignant cells revealed that a large fraction of phenotypic heterogeneity arises due to homeostatic stem and differentiation programs in cancer cells, with stem cells, TA, absorptive colonocytes and secretory cells observed (Figures 1C–1F and S1D–S1I).

In addition to the normal-like states, we also identify cancer-specific cell states. Two states share an expression profile with revSCSs described in injury/regeneration as well as in pCRC^9^^,^^10^ (Figure 1E), hereafter referred to as regenerative cells (RECs). Gene enrichment analysis (GEA) revealed enrichment of prognostic gene signatures (epithelial high-risk [EpiHR]^14^ and one of the five clinically relevant CRC subsets [CRISB]^21^) and signatures derived from metastasis-initiating cells in mouse models (high-relapse cells [HRC] KRT20^−/+^)^14^ in the RECs (Figure 1E). Interestingly, a subset of RECs upregulates interferon-stimulated genes (ISGs) (Figures 1E, 1F, and S1J), suggestive of an ongoing inflammatory response (hereafter called inflammatory RECs [iRECs]). This suggests that T cell-derived interferon γ (IFN-γ) signaling may activate an inflammatory response in cancer cells that are in proximity and that disseminated cells may escape immune attack through the expression of immunomodulatory molecules.^22^ (i)RECs upregulate regenerative signatures enriched in injury and repair, a fetal intestinal signature (*TACSTD2* and *ANXA1*), as well as yes-associated protein (YAP) target genes (Figures 1E and S1G; Table S2). They also show epithelial-to-mesenchymal transition (EMT) signatures but lack expression of mesenchymal markers and EMT transcription factors (TFs) (Figure S1K), indicating that (i)RECs are in a partial EMT (pEMT) state, maintaining their epithelial identity.

Among the cancer-specific states, we identified a stem-like state absent in the healthy colon, characterized by upregulation of WNT antagonists, including *NOTUM*, *NKD1*, and *APCDD1* (Table S1). Apc-mutant stem cells have been shown to secrete WNT inhibitors such as NOTUM to outcompete wild-type stem cells by driving their differentiation, thereby facilitating the outgrowth of Apc-mutant clones and development of premalignant adenomas.^23^ In line with this, our analysis shows that the stem NOTUM state is enriched in patients with *APC* mutations (Figure S2A). In addition, we identified a hypoxic state and a human leukocyte antigen (HLA)-high state. Interestingly, we also observed an intermediate state expressing both (i)REC and stem markers, potentially indicating a hybrid transition state between (i)REC and stem-like states. The intermediate state upregulates chemokines such as *CXCL2* and *CXCL3* (Figure 1F). All cancer cell states were present in all 4 datasets analyzed, with the exception of rarer tuft cells, which were not observed in one of the datasets (Figure S1H).

Individual tumors display extensive heterogeneity in the composition of cancer cell states (Figures 1D and S1H). To investigate this further, we first compared the mismatch repair-deficient (MMRd) and -proficient (MMRp) tumors. We find that the HLA-high state is more abundant in MMRd tumors, whereas stem, stem NOTUM, intermediate, and tuft cell states are more abundant in MMRp tumors^24^ (Figures S2B–S2D). Consistent with this, expression of cancer state signatures (Table S3) in bulk tumors reveals higher expression of an HLA-high signature in microsatellite instability-high (MSI-H) tumors (but not microsatellite instability-low [MSI-L] tumors) and lower expression of stem NOTUM, stem, and intermediate signatures in MSI-H tumors (Figure S2E). Moreover, MMRp tumors are enriched for the intrinsic consensus molecular subtype 2 (iCMS2) signature compared to MMRd tumors, which have higher iCMS3^25^ (Figure S2F).

Classifying tumors using consensus molecular subtypes (CMSs)^26^^,^^27^ showed that CMS1 tumors were enriched for T cells, while CMS4 tumors were enriched for myeloid and stromal cells (Figures S2G and S2H), consistent with previous studies. As expected, stem and stem NOTUM states were more abundant in CMS2 tumors (Figures 1G and S2I). RECs were enriched in both the immune-rich CMS1 and stromal-rich CMS4 tumors, whereas iRECs were enriched in CMS1 tumors, further implicating upregulation of the inflammatory signature as a result of interactions with the immune system. These observations were confirmed in bulk RNA-seq samples (Figure S2J; Table S3). (i)RECs were present across all tumor stages (Figure S2K). Overall, our results show that genetically and transcriptionally distinct tumor subtypes display different compositions of cancer states, with RECs more abundant in CMS1 and CMS4 subtypes.

### Cancer cell states in LM mimic cell states in primary CRC

**Figure 2:**
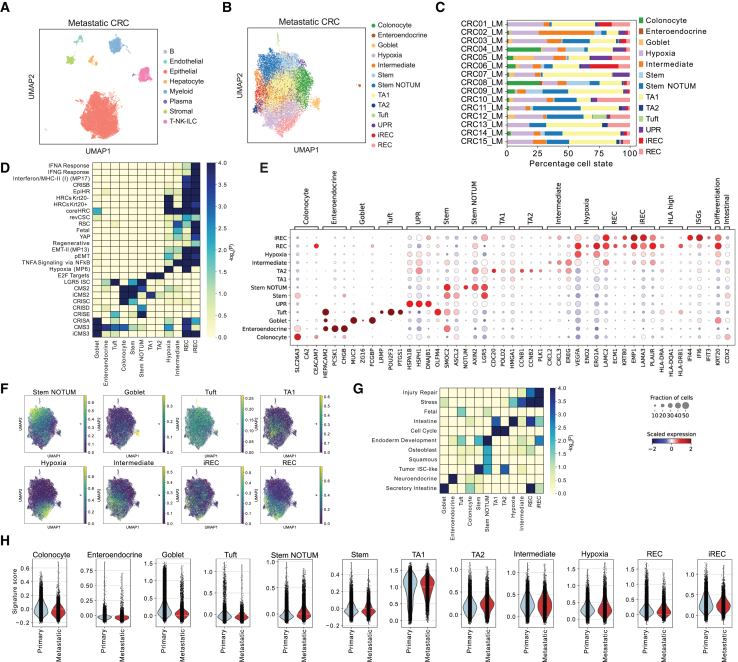
Cancer cell states are re-established in liver mCRC (A) UMAP representation showing cell types in mCRC Multiome data. (B) UMAP representation showing cancer cells in mCRC. (C) Proportion of cancer states across mCRC samples. (D) GEA of DEGs in mCRC states for the indicated signatures. (E) Scaled mRNA expression of indicated marker genes, ISGs, and *KRT20* in mCRC cell states. (F) pCRC signature scores in mCRC cells. (G) Heatmap showing GEA of mCRC DEGs in the indicated hotspot and fetal signatures.^15^ (H) Multiome mCRC state scores in primary and metastatic samples from Moorman et al.^15^

Next, we sought to investigate how the phenotypic heterogeneity in LMs differs from primary CRC. To characterize the regulatory networks that drive the different cancer states, we generated sn-Multiome data from 15 LMs, simultaneously profiling both mRNA and chromatin accessibility. Seven of the 15 patients had received chemotherapy prior to surgical resection (Table S4). After quality control, we retained 21,354 cells, including malignant, T, myeloid, stromal, and endothelial cells as well as liver-specific hepatocytes and cholangiocytes (Figures 2A and S3A–S3E). Uniform manifold approximation and projection (UMAP) representation based upon the ATAC modality gave similar results (Figure S3B). The majority of cells were epithelial (Figures S3C and S3D), consistent with published CRC snRNA-seq datasets.^28^

We isolated and analyzed the transcriptome of the malignant cells, revealing a structure surprisingly similar to pCRC (Figures 2B–2F and S4A–S4E). This suggests that, upon disseminating to the liver, metastasis-initiating cells can recreate the primary tumor structure at distant sites, consistent with observations in mouse models. Cancer cell states were largely conserved between treatment-naive and treated patients (Figures S4F and S4G). Overall, the cancer states are present in all LMs (with exceptions for rarer states such as tuft and enteroendocrine), but individual tumors show variation in their proportions (Figure 2C). Similar to pCRC, poor prognosis-associated EpiHR and CRISB signatures are enriched in both iREC and REC, whilst INF-α/γ hallmarks and ISGs are upregulated in iRECs (Figures 2D, 2E, and S4E). In addition, (i)REC states lack expression of mesenchymal markers but express pEMT genes (Figure S4D; Table S5). In both pCRC and metastatic CRC (mCRC), ISGs are enriched in iRECs, while hypoxia is enriched in RECs (Figures 1E, 2D, and S4H). Elevation of INF-α/γ hallmarks has also been detected in EMP1^+^ cells in micrometastatic lesions in a mouse CRC model.^16^ Analysis of paired snATAC-seq data show a similar chromatin landscape in iRECs and RECs, with only 39/20 open/closed differentially accessible regions (DARs) (Figure S4I). We did not observe an HLA-high cluster in LMs, but this could be due to the smaller dataset or lower proportion of MMRd samples in the dataset.

To confirm these results, we reanalyzed a recent study of matched normal colon, primary, and metastatic tissue,^15^ showing the same cell states as described in our Multiome dataset (Figures S5A–S5E). In addition to the canonical intestinal-like states, Moorman et al. revealed non-canonical cancer states enriched in rare chemo-treated metastatic samples, principally neuroendocrine-like and squamous-like states, as well an endoderm-like intermediate state that lies between canonical and non-canonical states.^15^ Comparison with our analysis shows that the injury repair signature maps to our (i)REC states (Figures 2G and S5E). The neuroendocrine signature was enriched in our enteroendocrine state; however, it was difficult to distinguish between both based on *CDX2* expression. Our enteroendocrine cells have lower *CDX2* expression compared to other cancer states in both pCRC and mCRC (Figure S4B) as well as in healthy colon (Figure S1E). The squamous signature was partially enriched in our stem NOTUM state, but no expression of the squamous markers *KRT5* and *KRT31* markers was observed (Figure S5D), potentially due to the enrichment of treatment-naive samples in our dataset. The squamous signature shares some WNT inhibitors, such as *NOTUM*, with the stem NOTUM state (Table S2; S5); however, in the Moorman et al. dataset, the squamous and our stem NOTUM signature map to separate clusters, suggesting that the stem NOTUM is distinct to the squamous state but potentially related (Figures S5B and S5C). Importantly, this analysis shows that our cancer cell state signatures are present in both primary and liver metastatic samples (Figure 2H).

As an additional validation, we also integrated and analyzed published LM scRNA-seq data from 17 patients^19^^,^^29^^,^^30^^,^^31^ and showed the same cell states as described in our previous analysis. A squamous-like state as described in Moorman et al. (*KRT5* and *KRT31*) was detected in one chemo-treated patient (Figures S5H and S5I). Interestingly, similar to our dataset, both the squamous and endoderm signatures were enriched alongside the stem NOTUM signature, suggesting that this state is potentially important for cellular transitions into non-canonical states. *PROX1*, identified as a lineage restriction regulator in injured epithelium,^15^ was also found upregulated in the stem NOTUM state (Figures S5D and S5I). Altogether, our analysis of LMs shows that the heterogeneous cancer cell states in primary CRC, including putative pro-metastatic RECs, mimic the cell states at distant liver metastatic sites.

### Transcription factors regulating malignant states

**Figure 3:**
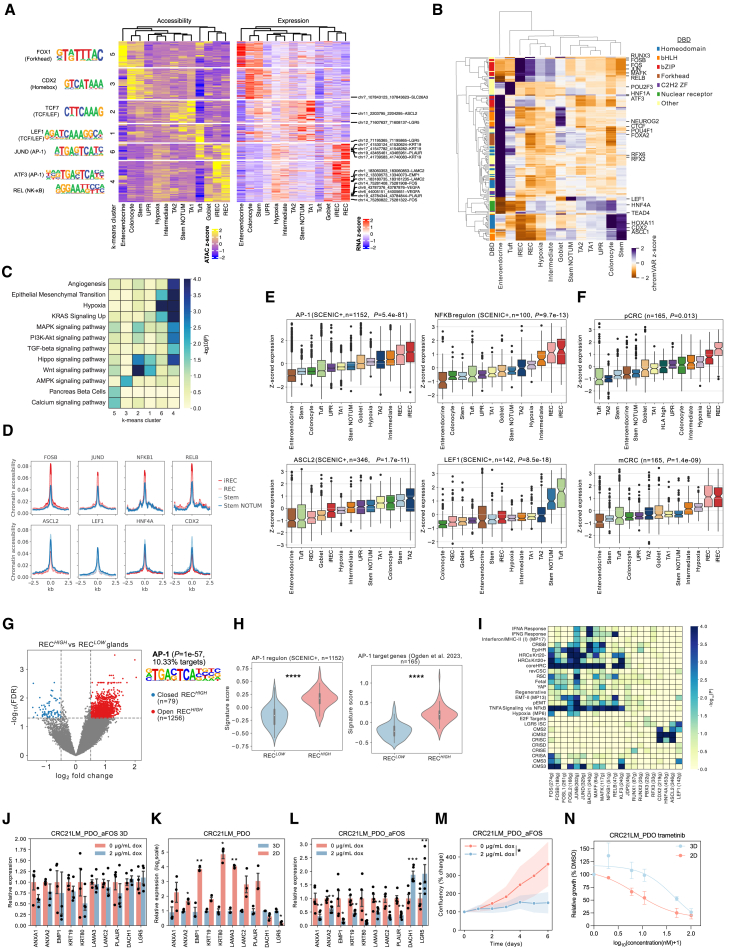
Regulation of cancer cell states (A) Left: chromatin accessibility of putative enhancers. Right: mRNA expression of genes linked to enhancers. (B) GEA of genes in k-means clusters using Kyoto Encyclopedia of Genes and Genomes (KEGG) pathways and MSigDB Hallmarks. (C) ChromVAR motif deviation *Z* scores for TFs in mCRC states (Wilcoxon, false discovery rate < 0.05), annotated based on the DNA binding domain.^116^ (D) Accessibility of chromatin regions in the indicated SCENIC+ regulons. (E) *Z*-scored expression of genes in SCENIC+ regulons across mCRC states. AP-1 and NF-κB regulons were formed by combining the regulons of *FOS* and *JUN* family members and the NF-κB subunits. One-way ANOVA. (F) *Z*-scored mRNA expression of AP-1 target genes^44^ across pCRC and mCRC states. One-way ANOVA. (G) Differentially accessible chromatin regions in *REC*^HIGH^ relative to *REC*^LOW^ glands.^45^ (H) Expression of AP-1 regulon genes or AP-1 target genes^44^ in *REC*^HIGH^ and *REC*^LOW^ glands. Unpaired t test. (I) GEA of the indicated signatures in TF regulons. Numbers in brackets indicate the number of genes in the regulon. (J) RT-qPCR analysis of the indicated genes following GFP-aFOS induction by 2 μg/mL doxycycline treatment in 3D. Paired t test; no genes were significant; *n* = 4. (K) RT-qPCR analysis of parental CRC21LM_PDO in 3D or on collagen I-coated plates (2D). Paired t test, *n* = 3. (L) RT-qPCR analysis of the indicated genes following GFP-aFOS induction under 2D culture conditions. Paired t test, *n* = 5. (M) Time-lapse live-cell imaging showing the change in confluency of organoids in 2D following GFP-aFOS induction. 2-way ANOVA, *n* = 3. (N) Cell proliferation assay of CRC21LM_PDO treated with trametinib in 3D and 2D culture systems; *n* = 3. Data are represented as mean ± SEM. ∗*p* < 0.05, ∗∗*p* < 0.01, ∗∗∗*p* < 0.001, ∗∗∗∗*p* < 0.0001.

To predict transcriptional regulators of cancer cell states, we next investigated the accessible chromatin landscape in our mCRC dataset. Peaks were called in each cancer state, and a union peakset was formed of 82,491 accessible chromatin regions, the majority (80.7%) of which are distal to promoters (Figure S6A). To identify putative enhancers, we correlated the expression level of genes with the accessibility of distal chromatin regions and identified 1,444 putative enhancer-gene linkages (PE-GLs). k-means clustering of PE-GLs identified clusters associated with the different cancer states, indicative of epigenetic regulation (Figure 3A). More than 60% of the putative enhancers overlap with a set of predicted enhancers in CRC organoids^32^ (Figure S6B), demonstrating the validity of this approach. We identified cell-type-specific PE-GLs potentially driving the expression of marker genes for stem cells (*LGR5* and *ASCL2*), colonocytes (*SLC26A3*), (i)RECs (*EMP1*, *PLAUR*, and *LAMC2*), and hypoxia (*VEGFA*) (Figures 3A, 3B, S6C, and S6D). GEA of genes in the PE-GL clusters in (i)REC states (clusters 4 and 6) shows enrichment for hypoxia, EMT, mitogen-activated protein kinase (MAPK), phosphatidylinositol 3-kinase, and tumor necrosis factor alpha (TNF-α) signaling, while WNT signaling is enriched in stem NOTUM (clusters 1 and 2) cells (Figure 3C), supporting our transcriptomics data. Additionally, the prognostic signatures EpiHR and CRISB are enriched in genes belonging to clusters 4 and 6 (Figure S6C). Therefore, PE-GLs likely establish important gene expression programs in cancer cell states.

Transcription factors are key regulators of cell identity and function. To predict TFs driving the different cancer cell states, we identified differentially accessible TF binding motifs.^33^ Interestingly, hierarchical clustering of the cancer states by motif accessibility shows that the intermediate state clusters closely with hypoxia and has increased accessibility of TF motifs that are also accessible in (i)RECs and stem cells (Figure 3D). We confirmed this by looking at all differentially open chromatin regions in (i)RECs and stem/stem NOTUM cells, which are also found to be accessible in the intermediate and hypoxia states, suggesting that these may be plastic, transitioning states (Figure S6E).

To further nominate TFs driving changes in gene expression in different cancer states, we identified TFs with the highest correlation between their gene expression and the chromatin accessibility of their cognate motif (Figure S6F). Motifs enriched in differentiated cell states include POU2F3 (tuft), RFX3/6 (enteroendocrine), HNF4A (colonocytes), and CDX2 (stem and Colonocytes) (Figures 3D and S6G), identified previously to play a role in differentiation in the healthy colon.^34^^,^^35^^,^^36^^,^^37^^,^^38^ Among the TFs most correlated with motif accessibility are AP-1 family members (FOS, FOSB, FOSL1, FOSL2, JUNB, JUND), nuclear factor κB (NF-κB) subunits (NFKB1, NFKB2, and RELB) enriched in (i)RECs, and LEF1 enriched in stem NOTUM (Figures 3D, S6F, and S6G). AP-1 family members are regulated by MAPK signaling^39^ and T cell factor/lymphoid enhancer-binding factor (TCF/LEF) family members by WNT signaling,^40^ consistent with enrichment of MAPK or WNT signatures in genes associated with (i)REC or stem NOTUM states, respectively (Figure 3C). *De novo* motif enrichment analysis of PE-GLs also demonstrates enrichment of AP-1 and NF-κB in (i)RECs and TCF/LEF in stem NOTUM PE-GLs (Figure 3A; Table S6), showing important regulatory roles of these TFs.

To further investigate the gene-regulatory networks governing (i)RECs, we predicted both genes and accessible chromatin regions regulated by TFs in cancer cell states.^41^ Single-cell regulatory network inference and clustering (SCENIC+) identified regulons for 22 of 26 TFs (exceptions are NR5A2, NEUROG3, HNF1B, and RFX6) whose expression correlated with motif accessibility. Focusing on AP-1 and NF-κB subunits as potential drivers of (i)REC states, we find that accessible chromatin regions in FOSB, JUND, NFKB1, and RELB regulons are more accessible in (i)RECs relative to stem-like cells, which feature greater accessibility of ASCL2, LEF1, HNF4A, and CDX2 regulons (Figure 3D). In addition, *de novo* motif analysis of chromatin regions in the RELB regulon shows significant enrichment of the AP-1 motif (Table S7), suggesting that AP-1 and NF-κB cooperate to establish the (i)REC state. Importantly, chromatin regions in JUND and HNF4A regulons are bound by JUND and HNF4A in CRC cell lines (Figure S6H), indicating the validity of our approach. Interestingly, we also find TEAD1 enriched in (i)RECs and TEAD4 in the intermediate state (Figures S6I and S6J). YAP/TAZ (transcriptional coactivator with PDZ-binding motif) are co-factors for the transcriptional enhanced associate domain (TEAD) TF family, and studies have shown cooperation between YAP/TAZ/TEAD and AP-1 at enhancers in different contexts.^42^^,^^43^ This is in line with the upregulation of YAP target genes in iRECs.

To corroborate this, we assessed the expression levels of genes in AP-1, NF-κB, ASCL2, and LEF1 regulons across the cancer states. The AP-1 regulon significantly overlaps (*p* = 1.32e^−26^, hypergeometric test) with experimentally determined AP-1 target genes identified in another gastrointestinal cancer, esophageal adenocarcinoma^44^ (Table S7). AP-1 and NF-κB target genes are expressed highest in (i)RECs compared to LEF1 and ASCL2 target genes, which show highest expression in tuft and stem NOTUM, and TA2 and stem cells, respectively (Figure 3E). This indicates that stem NOTUM and stem states are regulated by different TFs, which is supported by expression levels of *ASCL2* and *LEF1* and accessibility of their binding motifs (Figure S6J).

Experimentally determined AP-1 target genes are also expressed at higher levels in (i)RECs in both pCRC and mCRC (Figure 3F), consistent across all 15 mCRC samples (Figure S6K). In addition, the (i)REC marker gene *EMP1* is within the FOS, FOSB, and JUNB regulons (Table S8), and in bulk pCRC RNA-seq data, *EMP1* expression correlates with these AP-1 members (Figure S6L), suggesting that (i)RECs are also regulated by AP-1 in pCRC. We confirmed this by analyzing snATAC-seq data from 4 primary CRCs (Figure S6M) as well as a large dataset of 196 single glands with paired RNA and ATAC-seq data from 26 patients,^45^ where we compared glands with a high (REC^*HIGH*^) and low (REC^*LOW*^) signature. We show significant enrichment of the AP-1 motif in chromatin regions differentially open in REC^*HIGH*^ glands and higher expression of AP-1 target genes (Figures 3G and 3H). GEA on genes within TF regulons shows that the LGR5 ISC signature is significantly enriched in ASCL2 and LEF1 regulons, while many gene signatures enriched in (i)RECs are also significantly enriched in AP-1 family members, including signatures associated with poor prognosis (CRISB and EpiHR) and metastasis-initiating cells (HRCs) (Figure 3I).

To validate AP-1 as a key driver of (i)REC cell states, we generated three patient-derived organoid lines (1 primary and 2 metastatic) containing an inducible dominant-negative AP-1 construct (GFP-aFOS).^46^^,^^47^ Inhibition of AP-1 had no effect on the expression of (i)REC markers under standard organoid culture conditions (3D) in 2 organoid lines (Figures 3J and S7A), potentially due to these conditions favoring stem-like cell states. Therefore, we explored a culture system to push cancer cells into an (i)REC state. A recent study reported induction of regenerative signatures using a 2D collagen I/IV/hyaluronan culture system.^48^ We therefore tested culturing organoids in 2D on collagen I-coated plates, which induced (i)REC marker genes, while repressing the stem cell markers *LGR5* and *DACH1* (Figures 3K and S7B), indicative of cancer cells transitioning into (i)REC states. In 2D culture, inhibition of AP-1 caused a marked decrease of several (i)REC markers and predicted AP-1 target genes (Figures 3L and S7). Additionally, AP-1 inhibition impaired the ability of organoid cells to grow in 2D culture (Figure 3M).

Signaling pathways affect TF activity, and MAPK signaling is a known regulator of AP-1 at the transcriptional and post-transcriptional level.^49^ GEA indicated activation of MAPK signaling in (i)REC cell states. To further explore this, we used The Cancer Genome Atlas Program (TCGA) reverse-phase protein array data, which indicated greater abundance of active MEK1 (MAP2K1), ERK1/2 (MAPK1/3), and p38 MAPK (MAPK14) in tumors with high expression of (i)REC signatures (Figure S7D). Organoids in 2D showed increased sensitivity to MEK inhibition using the US Food and Drug Administration (FDA)-approved inhibitor trametinib (Figures 3N and S7E), demonstrating that particular cancer states have specific therapeutic vulnerabilities. Collectively, this suggests that MAPK signaling is associated with (i)REC states.

### The landscape of the tumor microenvironment in primary and metastatic CRC

TME pressures can drive cancer cells to adapt to different conditions and acquire pro-metastatic traits for colonization of secondary organ niches. To better understand the extrinsic signals shaping distinct cancer states, we next analyzed the non-malignant cells in both pCRC^17^^,^^18^^,^^19^^,^^20^ (Figure S8) and mCRC (Figure S9). For comprehensive analysis of the mCRC TME, we integrated our Multiome data with published scRNA-seq data from 26 additional patients,^19^^,^^20^ generating a dataset containing 98,312 transcriptomes (Figures S9A–S9C).

Within the stromal subpopulations, CAFs and endothelial cells were the major cell types (Figures S8A, S8B, S9D, and S9E). Additionally, we find pericytes (*RGS5*), vascular smooth muscle cells (*MYH11*), and, in pCRC, also enteric glial cells (*PLP1*). The CAFs separate into inflammatory (iCAFs) (*CXCL12* and *C3*), ECM-remodeling (*POSTN* and collagen and matrix metalloproteinase genes), and contractile myofibroblasts (*ACTA2*) (Figures S8A and S9D). Furthermore, in pCRC, we observe fibroblasts present in the normal colon: BMP-producing CAFs (*CXCL14*), which drive differentiation of epithelial cells,^50^ and GREM1^+^ CAFs that produce stem cell niche factors such as *RSPO3.*^51^ The endothelial cells are divided into vascular stalk-like (*ACKR1* and *SELP*) and tip-like (*RGCC* and *KDR*) cells, lymphatic endothelial (*LYVE1*), and proliferating cells (Figures S8B and S9E). In mCRC, we also find liver-specific sinusoidal endothelial cells.

The myeloid compartment comprises tumor-associated macrophages (TAMs), monocytes, neutrophils, and dendritic cells (DC), as well as Kupffer cells in mCRC (Figures S8C and S9G). Within the TAMs, we identify immunosuppressive SPP1^+^ TAMs and C1QC^+^ TAMs, both upregulating lipid-laden signatures^52^ (Figures S8D and S9H). SPP1^+^ macrophages have been described as pro-metastatic and angiogenic TAMs potentially driven by hypoxia,^53^ whereas TAMs expressing *C1QC* and *TREM2* induce T cell exhaustion and regulatory T cells (Tregs) infiltration.^54^ However, a subset of C1QC TAMs also upregulates HLA-DR molecules, suggesting antigen presentation ability and potential anti-tumor functions.^55^ We also find IL1B^+^ and NLRP3^+^ subpopulations, characterized by high expression of an inflammatory signature (*CCL3* and *CXCL3*).^56^ IL1B^+^ macrophages were found colocalizing with EMT-enriched tumors cells at the invasive edge in kidney cancer,^57^ whereas in pancreatic cancer they have been shown to induce inflammatory reprogramming of cancer cells.^58^ The monocyte subsets include FCN1^+^CD14^+^ monocytes and intermediate CD16^+^CD14^+^ monocytes. The DCs separate into conventional cDC1 (*CLEC9A*) and cDC2 (*CD1C*), CCR7^+^LAMP3^+^ migratory DCs and LILRA4^+^ plasmacytoid DCs.

T lymphocytes comprise diverse CD8^+^ and CD4^+^ T cells, spanning from naive to effector to exhausted states (Figures S8F and S9J). We also detect two subsets of natural killer (NK) cells, distinguished by expression of *XCL1*, *XCL2*, and *GZMK* in NK1 and higher expression of granules, *KIRD2L1*, and *KIR3DL2* in NK2 as well as NK T, innate lymphoid cells (ILCs), and γδ T cells.

Additionally, in mCRC, we also recover hepatocytes (*APOA1*) and cholangiocytes (*SOX9* and *CLDN4*) (Figure S9F).

### RECs localize at the invasive tumor edge

**Figure 4:**
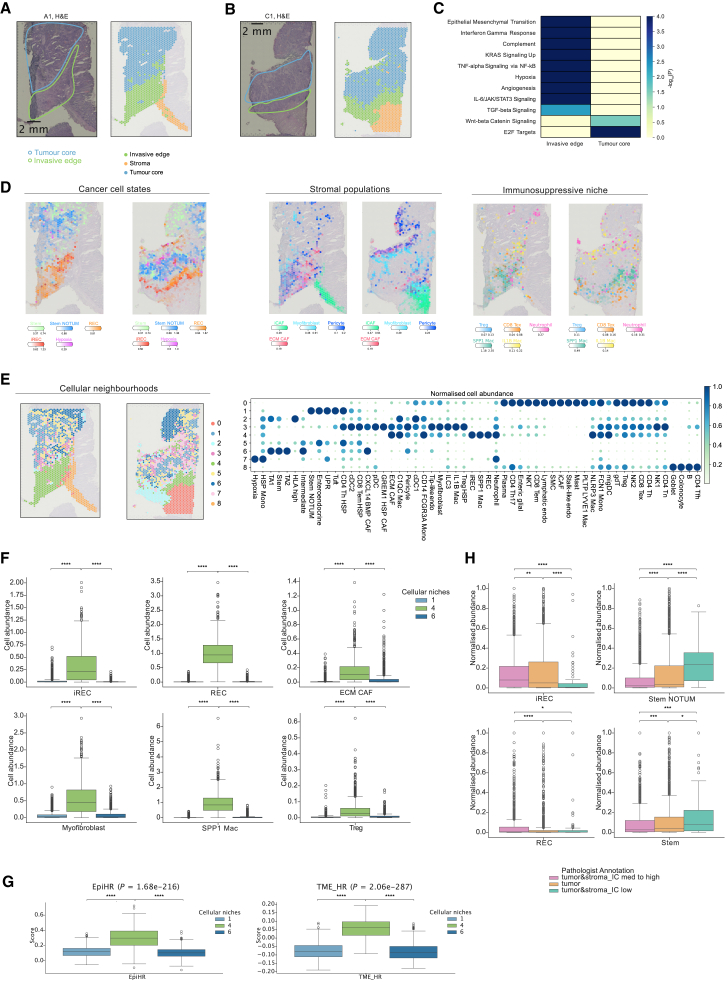
Spatial neighborhoods in pCRC (A) Left: H&E staining and pathologist annotations of sample A1.^20^ Right: clustering annotations. (B) Left: H&E staining and pathologist annotations of sample C1.^20^ Right: clustering annotations. (C) GEA of upregulated genes in the invasive edge and the tumor core. (D) Abundance (color represents intensity) of cancer, stromal, and immune subpopulations in samples A1 and C1. (E) Left: spatial neighborhoods in samples A1 and C1. Right: dot plot representing average cell abundance (dot size and color) for each cell state, per neighborhood, and normalized between 0 and 1 per cell state. (F) Abundance of relevant cell types across cellular neighborhoods of the invasive edge and tumor core. (G) Expression of EpiHR and TME-HR signatures^14^ in spots in the spatial neighborhoods. (H) Abundance of relevant cell states in 6 samples^63^ stratified by histopathological annotation. (F, G, H) Kruskal-Wallis test followed by post hoc Dunn test. ∗*p* < 0.05,∗∗*p* < 0.01, ∗∗∗*p* < 0.001, ∗∗∗∗*p* < 0.0001; Benjamini-Hochberg adjustment.

To dissect the spatial organization of CRC tumors, we analyzed published spatial Visium data from 4 pCRC samples.^59^ The tumor core and invasive edge annotations from the original publication were confirmed by manual assessment of the hematoxylin and eosin (H&E) staining (Figures 4A and 4B). GEA of differentially expressed genes (DEGs) between the tumor core and invasive edge (Table S8) reveals that, while cell cycle and WNT signaling pathways are enriched among upregulated genes in the tumor core, the invasive edge is enriched with EMT, hypoxia, IFN-γ response, NF-κB, angiogenesis, and Kirsten rat sarcoma virus (KRAS) signaling (Figures 4C, S10A, and S10B), further indicating that MAPK signaling may drive transitions into (i)REC states at invasive fronts.

Next, we spatially mapped the fine-grained cell types/states defined by scRNA-seq data onto their spatial location using cell2location.^60^ We then used SpatialDE2^61^ to identify cellular niches across all samples. Interestingly, our findings reveal spatial localization of the (i)RECs at the invasive tumor edge (neighborhood 4; Figures 4D–4F, S10C, and S10D), further supporting the hypothesis that these states are putative metastasis-initiating cells, as shown previously in mouse CRC models.^14^ In comparison, the stem cells are abundant at the tumor core (neighborhoods 1 and 6). (i)RECs at the invasive edge colocalize with myofibroblasts and ECM CAFs as well as immunosuppressive cells such as SPP1^+^ macrophages, neutrophils, CD8^+^ exhausted T (Tex) cells, and Tregs (Figures 4E, 4F, S10D, and S10E). (i)RECs are also in close proximity to perivascular cells, suggesting a connection with hematogenous or lymphatic dissemination. Near the border between the invasive edge and healthy colon, we also find an abundance of inflammatory iCAFs known to be involved in recruitment and polarization of immunosuppressive myeloid cells.^62^ Signatures associated with poor prognosis (epithelium specific, EpiHR; TME specific, TME HR)^14^ are upregulated in the (i)REC neighborhood compared to others (Figure 4G). Non-negative matrix factorization (NMF) analyses confirmed the colocalization of (i)RECs with CAFs and immunosuppressive cells (factor 2; Figure S10F).

Separate analysis of 3 Visium pCRC samples^29^ corroborated our findings, depicting a similar cellular neighborhood of (i)RECs with neutrophils, SPP1 macrophages, ECM CAFs, and Tregs (neighborhood 1; Figures S11A–S11D). We further validated our results using 6 additional Visium samples,^63^ which were annotated into distinct anatomical compartments based on tissue type. We find that iRECs are significantly enriched in the tumor-stroma-immune interface compared to stem populations, which are more abundant in the tumor and the tumor-stroma low regions (Figure 4G). Finally, we extended these observations to a larger cohort by analyzing TCGA RNA-seq data from 609 patients. These results show high correlation of iREC and immune scores in contrast to stem signature scores (Figure S11E), further supporting the effect of proximity of immune cells to activation of inflammatory signatures in iRECs.

### Cancer and TME cells organized in cellular niches in LM

Next, we sought to interrogate how cancer states and the TME interact in LM compared to pCRC. To this end, we generated Visium data from three LM samples for which we had paired Multiome data and additionally analyzed published Visium data of three LM samples.^20^

**Figure 5:**
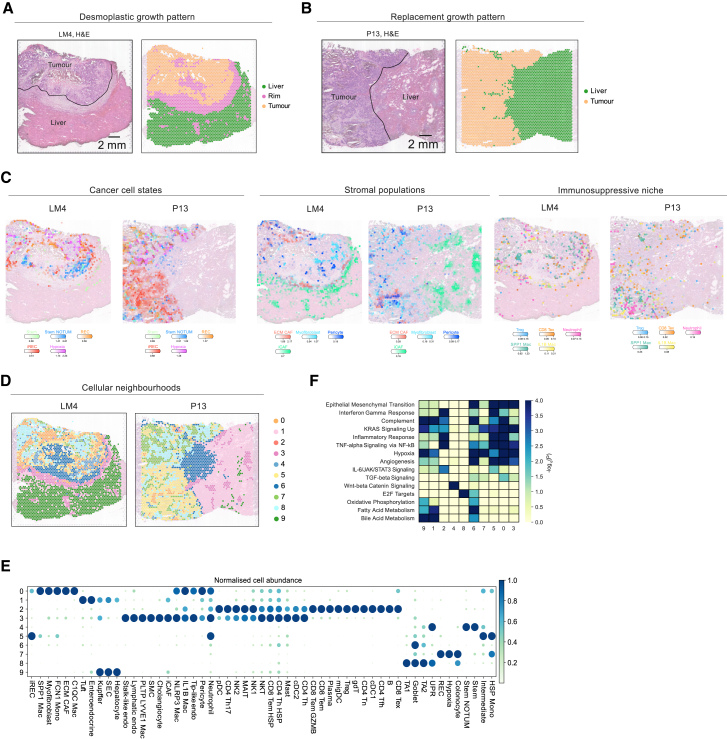
Spatial cellular niches in liver mCRC (A) H&E staining of representative sample LM4^20^ (neoadjuvant chemotherapy), manual annotations, and clustering annotations. The dashed line denotes the desmoplastic rim. (B) H&E staining of sample P13^20^ (untreated), manual annotations, and clustering annotations. The dashed line denotes the tumor-liver border. (C) Abundance (color represents intensity) of cancer, stromal, and immune subpopulations in samples LM4 and P13. (D) Spatial cellular neighborhoods in LM4 and P13. (E) Dot plot representing average cell abundance (dot size and color) for each cell state, per neighborhood, and normalized between 0 and 1 per cell state. (F) GEA of upregulated genes in the cellular neighborhoods.

LMs exhibit distinct histological growth patterns, reflecting different ways in which cancer cells interact with the surrounding liver parenchyma.^64^ The desmoplastic growth pattern (sample LM4) is characterized by a desmoplastic capsule that consists of fibroblasts and extracellular matrix, effectively encapsulating the metastatic cancer tissue from the liver (Figures 5A and 5C). In contrast, the cancer cells in the replacement growth pattern (sample P13) are in direct contact between hepatocytes (Figures 5B and 5C). This growth pattern is also characterized by very low T cell infiltration (Figures 5C and S12A–S12C). Across all three samples capturing the tumor site and liver parenchyma (LM4, P13, and P3), we detect a layered spatial organization of stromal and myeloid cells from the liver parenchyma to the central tumor stroma (Figures 5C and S12A–S12C). iCAFs are enriched at the liver site and the tumor-liver interface, ECM CAFs with C1QC^+^ and inflammatory IL1B^+^ macrophages at the tumor-liver border and in the tumor site, whereas myofibroblasts and immunosuppressive SPP1^+^ macrophages infiltrate the tumor core.

Joint analysis across all six LM samples shows spatially segregated cancer states organized into distinct local neighborhoods (Figures 5D, 5E, and S12D). The iRECs, similar to the invasive edge in pCRC, congregate with perivascular cells, ECM remodeling CAFs, and myofibroblasts in an immunosuppressive niche comprising SPP1^+^ TAMs, IL1B^+^/NLRP3^+^ TAMs, neutrophils, exhausted CD8^+^ T cells, and Tregs (neighborhoods 0 and 5 and low abundance in neighborhood 3). The stem cells reside in their own niche; however, they also colocalize with the intermediate state (neighborhood 4), implicating potential transitions between stem NOTUM and intermediate cells. Interestingly, in contrast to pCRC, the REC subpopulation is separate from iRECs and immune cells and colocalizes with the hypoxia state (neighborhood 7).

Although cellular neighborhoods are often shared among samples, we also capture spatial features that highlight inter-patient heterogeneity (Figure S12E). Cellular neighborhoods 1 and 9 denote the liver parenchyma, primarily composed of hepatocytes and Kupffer cells, and they are specific for the replacement and desmoplastic patterns, respectively. Neighborhood 3 comprises immunomodulatory CAFs and immunosuppressive cells and is mostly enriched at the desmoplastic rim, whereas neighborhood 2 is largely composed of immune cells and specific to samples CRC11 and CRC09 (Figures 5D, 5E, S12D, and S12E). However, all neighborhoods are present in both chemo-treated and treatment-naive samples (Figure S12G).

To highlight the distinct spatial transcriptional features that different cellular niches have, we performed differential expression analysis between the cellular neighborhoods (Table S5). GEA confirms that genes upregulated in iREC- and immune-enriched neighborhoods (neighborhoods 0, 2, 3, and 5) are enriched for EMT, IFN-γ response, KRAS signaling, and angiogenesis, whereas the stem-enriched niche (neighborhood 4) is enriched for the WNT signaling pathway (Figure 5F). Consistent with this, EMT and IFN response signature scores are higher in the iREC and immune-enriched niches (Figures S12H–S12J). Conversely, they are inversely related to WNT signaling, which is higher in the stem-enriched niche. NMF analysis confirmed the colocalization of iRECs with SPP1^+^, C1QC^+^, and IL1B^+^ TAMs, CD8 Tex cells, myofibroblasts, ECM CAFs, and pericytes (fact_8; Figure S12K). Furthermore, the stem NOTUM cells congregate with the intermediate state (fact_3), whereas the RECs are in close proximity to hypoxia (fact_5), supporting our findings.

We further validated these results using five additional formalin-fixed paraffin-embedded published Visium LM samples.^65^ Consistent with our previous results, iRECs reside in an immunosuppressive niche (neighborhoods 1 and 5) surrounded by myofibroblasts; pericytes; neutrophils; IL1B^+^, NLRP3^+^, and SPP1^+^ macrophages; and exhausted CD8^+^ T cells and Tregs (Figures S13A–S13C).

Overall, our observations suggest that specific subtypes of CAFs, macrophages, and exhausted CD8 T cells mediate the phenotype of the iRECs both in primary CRC and LM.

### Cancer-TME interactions mediate regenerative inflammatory cells

Given the close proximity of CAFs and TAMs with iRECs in both primary and LM, we next investigated potential mediators of cellular crosstalk between these compartments. We first used CellPhoneDB^66^ to identify enriched receptor-ligand pairs among the cell states residing in the spatial niche surrounding iRECs. Next, to determine which ligands potentially promote the inflammatory regenerative program in cancer cells, we identified ligands predicted to induce the AP-1 and NF-κB regulons using NicheNET.^67^

**Figure 6:**
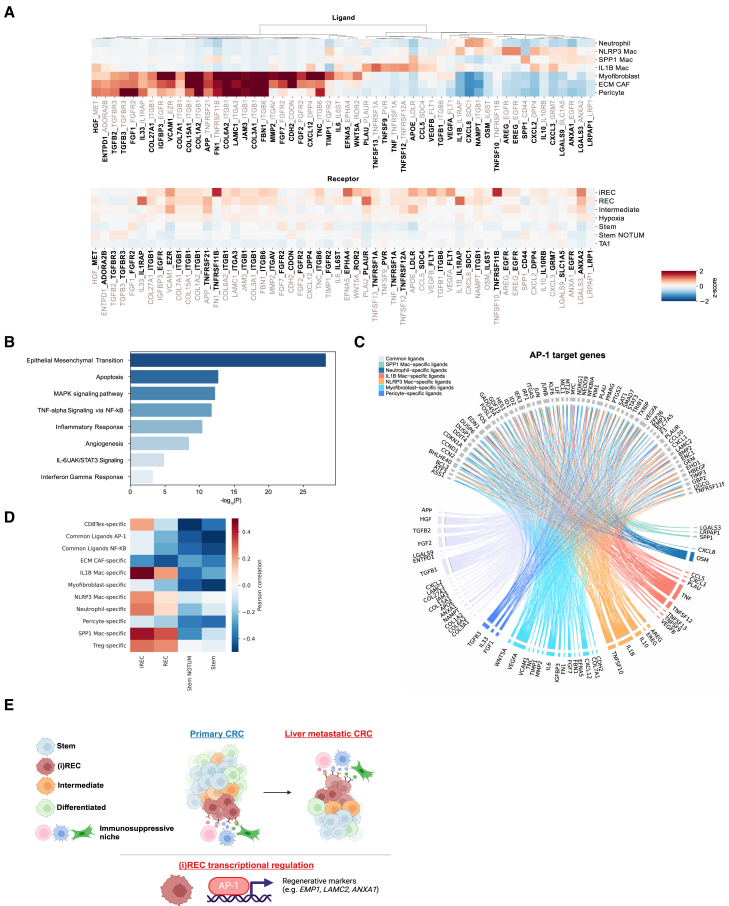
Cellular interactome of iRECs (A) Heatmaps showing *Z* score expression of selected ligands in TME cells (top) and corresponding receptors in cancer states (bottom). Shown are ligands in bold and receptors in gray for senders (top) and opposite for receivers (bottom). (B) GEA of ligands predicted to activate the AP-1 regulon in iRECs. (C) Circos plot depicting links (regulatory potential scores) between predicted ligands and AP-1 target genes. (D) Correlation of the expression of the pCRC state signatures and ligands predicted to activate AP-1 and NF-κB regulons in TCGA CRC RNA-seq data. Common are ligands whose expression is shared in more than one subpopulation. (E) Graphical summary created in BioRender.

Our results reveal a candidate list of CAF- and myeloid-derived ligands potentially activating AP-1 target genes, with corresponding receptors upregulated in the (i)RECs (Figure 6A). GEA of the predicted ligands shows enrichment of EMT, MAPK signaling, inflammatory response, and IFN-γ response, processes that are activated in iRECs (Figure 6B). Several ligands expressed in ECM CAFs, myofibroblasts, and pericytes have established roles in inducing EMT, invasion, and immune evasion, including transforming growth factor β (TGF-β) (*TGFB2*/*3*), *HGF*, fibroblast growth factors (*FGF1*/*2*/*7*), VEGF (*VEGFA/B*), and *IL6*. In addition, ECM CAFs and myofibroblasts are likely involved in matrix remodeling through secretion of matrix metalloproteinases (*MMP2*) and collagens.^68^ *IL-33*, which has been shown to activate and maintain immunosuppressive TAMs^69^^,^^70^ and CD39 (encoded by *ENTPD1*), which, together with CD73 convert ATP to adenosine to prevent immune activation,^69^ are also candidate CAF-secreted ligands. Cognate receptors for these ligands are expressed higher in (i)REC states compared to other cancer states as well as in (i)REC^*HIGH*^ single glands (Figures 6A and S14A).

In addition to stromal cells, myeloid and T cells are also predicted to induce an inflammatory phenotype in iRECs through activation of NF-κB, driven by proinflammatory genes such as IFN-γ and *CCL5* expressed in exhausted T cells as well as *IL1B* and *TNF* expressed in inflammatory TAMs (Figure S14B). Furthermore, *APOE* is highly expressed in SPP1^+^ and IL1B^+^ TAMs and has been shown to induce expression of immunosuppressive factors such as *CXCL1* and *CXCL5*,^71^, whereas *ANXA1* has been implicated in promoting immune suppression^72^ and resistance to chemotherapy in CRC.^73^ Other predicted ligands include chemokines such as CXCL2 and CXCL3 that can recruit neutrophils, contributing to establishment of an immunosuppressive environment.^74^

Spatial transcriptomics data show spatial enrichment of the predicted ligands in the cellular neighborhoods surrounding iRECs (Figure S14C), further supporting our findings. To extend these observations to a larger population, we analyzed TCGA RNA-seq data from 609 pCRC samples, showing high correlation of expression of IL1B^+^ macrophage- and exhausted CD8 T cell-derived ligands with the (i)REC signature (Figure 6D).

Collectively, these results highlight ligands predicted to induce a regenerative and inflammatory phenotype in iRECs and could lead to potential therapeutic strategies by targeting specific molecular mechanisms in the cellular niche that sustains the iREC state.

## Discussion

To form metastases, cancer cells undergo phenotypic transitions to leave the primary site, survive in the circulation, adapt to new microenvironments, and regenerate tumors at distant sites. Cellular plasticity provides cancer cells access to developmental or regenerative programs to adapt to new environments.

Using single-cell multiomics data from patients with primary CRC and LM, here we show that the heterogeneous malignant cell states in pCRC are re-established in metastasis. We reveal states with regenerative and inflammatory phenotypes that closely resemble metastasis-initiating cells in mouse models. We identify the transcription factors AP-1 and NF-κB as their regulators, suggesting that pathways regulated by those TFs, such as MAPK signaling, can serve as potential therapeutic targets to eliminate (i)RECs. AP-1 and NF-κB have also been implicated as transcriptional regulators in fetal intestinal organoids,^75^ supporting emerging evidence that cancer progression often requires reacquisition of developmental programs.

Upregulation of inflammatory genes suggests that interactions with immune cells may activate this response in cancer cells in close proximity. Treatment with neoadjuvant immunotherapy in mouse models suggest that iRECs could potentially be more sensitive to it^14^; however, prolonged IFN signaling can promote resistance associated with cancer cells epigenetically acquiring immunological memory that promotes immune dysfunction.^22^ Interestingly, AP-1 has been shown to have a crucial role in mediating epigenetic inflammatory memory.^76^ Therefore, future efforts using a combination of immunotherapy and blocking IFN-I signaling should be explored.

The existence of a hybrid intermediate state expressing both REC and stem markers indicates cellular transitions, presenting a significant challenge for targeting either population. Our patient-derived data are therefore in agreement with mouse studies demonstrating that the majority of metastases are seeded by LGR5^−^ cells that are able to transition back to LGR5^+^ cells and re-establish the primary cellular heterogeneity to form metastases. Interestingly, we also reveal a novel stem-like state, characterized by upregulation of WNT antagonists (NOTUM), that is potentially related to the squamous-like non-canonical state enriched in rare chemo-treated samples. This indicates that, in the dynamic intestinal epithelium, cellular transitions are complex, and plasticity may go beyond the stem-to-regenerative transition.

We reveal spatially segregated cancer states organized into neighborhoods with local cell-cell interactions, pointing to the role of the microenvironment in mediating and sustaining distinct cancer subpopulations. We find that the phenotype of iREC cells is likely maintained by specific TME subpopulations, including ECM CAFs and immunosuppressive and inflammatory myeloid and CD8^+^ T cells. Additionally, our analysis shows a spatial organization specific to distinct metastatic growth patterns, with clear differences between desmoplastic and replacement growth patterns. Considering that different histological growth patterns are prognostic markers for patient outcome and might be associated with different immune responses and mechanisms of treatment,^77^ our results emphasize a need to further investigate how these growth patterns arise.

Non-genetic plasticity plays a crucial role in CRC initiation, progression, metastasis, and resistance to therapy and represents a formidable challenge in cancer therapy. By identifying and characterizing the distinct malignant states, their regulatory drivers and microenvironmental cues that maintain them in primary and metastatic CRC, our findings might lead to novel therapeutic opportunities to impair plasticity by restricting transitions into invasive regenerative phenotypes.

### Limitations of the study

Although our study leverages spatial data to show localization of iRECs in an immunosuppressive niche, it lacks single-cell resolution, and we relied on deconvolution to infer cell-specific gene expression. We used multiple datasets to confirm our conclusions; however, further studies should validate this using higher-resolution spatial techniques. The candidate ligand-receptor interactions that potentially activate the AP-1 or NF-κB networks could be further validated using perturbation of PDOs. Last, due to the smaller number of LM samples, the majority of which are treatment naive, we did not detect rare non-canonical states as described previously.^15^

## Resource availability

### Lead contact

Further requests for resources should be directed to the lead contact, Mirjana Efremova (m.efremova@qmul.ac.uk).

### Materials availability

This study did not generate new unique reagents.

### Data and code availability

Sequencing data have been deposited at ArrayExpress (E-MTAB-13651, E-MTAB-13652 and E-MTAB-13655). Processed data are available at Mendeley Data (https://doi.org/10.17632/yd7bb3shn5.1) and the code at https://github.com/EfremovaLab/CRC_metastasis.

## Consortia

The members of the Cancer Tissue Bank are Claude Chelala, Dayem Ullah, Jo-Anne ChinAleong, and Amina Saad.

## Acknowledgments

We are grateful to members of the Cancer Tissue Bank and consultant surgeons (Ajit Abraham, Deepak Hariharan, and Vincent Yip). We are grateful to Dr. Hans Clevers (Roche) for sharing CRC organoids, Prof. Chris Tape and his team (UCL Cancer Institute) for help with establishment of LM organoids, and Prof. Andrew Sharrocks for the plasmid pINDUCER20-GFP-aFOS. We thank the UCL Single Cell Genomics Facility and the 10.13039/501100019154BCI Pathology and Microscopy Facility. M.E. was supported by the Bart Charity (MGU045), a 10.13039/501100000289Cancer Research UK (CRUK) career establishment award (RCCCEA/100003), and 10.13039/501100000289CRUK City of London (C7893/A26233). The Cancer Tissue Bank is supported by a 10.13039/501100000289CRUK Centre of Excellence award. The Single Cell Genomics Facility is supported by 10.13039/501100000289CRUK City of London (C7893/A26233). This research utilized the Apocrita HPC facility, supported by QMUL Research-IT.

## Author contributions

Conceptualization, M.E.; methodology, S.O., N.M., and M.E.; investigation, S.O., N.M., O.L., A.M.B, A.-M.B., M.B., M.G., T.G., H.M.K., and M.E.; computational analysis, S.O., N.M., E.A.S., and M.E.; writing – original draft, S.O., N.M., and M.E.; writing – review and editing, all authors.

## Declaration of interests

The authors declare no competing interests.

## STAR★Methods

### Key resources table

**Table:** 

REAGENT or RESOURCE	SOURCE	IDENTIFIER
**Antibodies**		

phospho-p44/42 MAPK primary antibody	Cell Signaling Technology	RRID: AB-2315112
p44/42 MAPK primary antibody	Cell Signaling Technology	RRID: AB-330744
HRP-linked secondary antibody	Cell Signaling Technology	RRID: AB-2099233

**Biological samples**		

Colorectal cancer liver specimens	Barts Cancer Tissue Bank; www.cancertissuebank.org	N/A

**Chemicals, peptides, and recombinant proteins**		

Advanced DMEM/F-12	Thermo Fisher Scientific	Cat#11540446
L-Glutamine	Thermo Fisher Scientific	Cat#25030081
Penicillin/streptomycin	Thermo Fisher Scientific	Cat#15140122
N-acetyl-L-cysteine	Sigma-Aldrich	Cat#A9165
HEPES	Thermo Fisher Scientific	Cat#11560496
B-27	Thermo Fisher Scientific	Cat#17504044
N2	Thermo Fisher Scientific	Cat#17502048
EGF	Thermo Fisher Scientific	Cat#PMG8041
Gastrin I	Sigma-Aldrich	Cat#SCP0152
A83-01	CliniSciences	Cat#04-0014
SB202190	Cambridge Bioscience	Cat#10010399
Nicotinamide	Sigma-Aldrich	Cat#N0636
Collagenase IV	Thermo Fisher Scientific	Cat#17104019
Primocin	InvivoGen	Cat#ant-pm-1
gentleMACS™ C tube	Miltenyi Biotec	Cat#130-093-237
ACK lysis buffer	Thermo Fisher Scientific	Cat#A1049201
Cultrex Reduced Growth Factor Basement Membrane Extract, Type 2, Pathclear (BME2)	Biotechne	Cat#3533-005-02
TrypLE™	Thermo Fisher Scientific	Cat#12604013
Cellmatrix Type I -A	Nitta Gelatin	Cat#631-00651-FJ
Y-27632	MedChem Express	Cat#HY-10071
Polybrene	Merck Millipore	Cat#TR-1003-G
G418	Thermo Fisher Scientific	Cat#11811023
Doxycycline	Cayman Chemicals	Cat#14422
Murine WNT3A	Peprotech	Cat#315-20
Murine Noggin	Peprotech	Cat#250-38
Human R-spondin-1	Peprotech	Cat#120-38
Trametinib	MedChem Express	Cat#HY-10999
Tris-HCl pH 7.4	Sigma-Aldrich	Cat#T2194
SDS	Sigma-Aldrich	Cat#436143
Glycerol	Sigma-Aldrich	Cat#G7757
EDTA	Thermo Fisher Scientific	Cat#10031660
Sodium Fluoride	Sigma-Aldrich	Cat#S1504-100G
β-glycerophosphate	Sigma-Aldrich	Cat#G9422
Phenylmethanesulfonyl Fluoride	Sigma-Aldrich	Cat# P7626
Sodium Orthovanadate	Thermo Scientific Chemicals	Cat#10114740
Protease inhibitor tablet	Roche	Cat#11836170001
Phosphatase inhibitor tablet	Roche	Cat#4906837001
0.2-μm multi-well filter plate	Cytiva	Cat#5053
2-well bis-tris gels	Invitrogen	Cat#15324604
Nitrocellulose membranes	Bio Rad	Cat#1620115
Intercept Blocking Buffer	LI-COR	Cat#927-60001
Tween 20	Sigma-Aldrich	Cat#P1379
Chemiluminescent substrate	Themo Scientific	Cat#34580
Re-blot solution	Millipore	Cat#2504
EZ lysis buffer	Sigma-Aldrich	Cat#NUC101-1KT
Sodium Chloride	Sigma-Aldrich	Cat#S9625
Calcium Chloride	Thermo Fisher Scientific	Cat#C-1400-53
Magnesium Chloride	Sigma-Aldrich	Cat#M1028
MACS BSA Stock Solution	Miltenyi-Biotec	Cat#130-091-376
RNase inhibitor	Merck	Cat#3335399001
DTT	Sigma-Aldrich	Cat#646563
Digitonin	Thermo Fisher Scientific	Cat#BN2006
Nuclei buffer	10× Genomics	Cat#2000153
Ethidium homodimer-1	Thermo Fisher Scientific	Cat#E1169

**Critical commercial assays**		

RNeasy Plus Mini Kit	Qiagen	Cat#74136
Luna® Universal One-Step RT-qPCR Kit	New England Biolabs	Cat#E3005
CellTiter-Glo® 3D assay	Promega	Cat#G9681
BCA assay	Thermo Fisher Scientific	Cat#A55864
Chromium Next GEM Single Cell Multiome ATAC + Gene Expression Reagent kits	10× Genomics	Cat#1000285
Chromium Next GEM Chip J Single Cell Kit	10× Genomics	Cat#1000234
Visium Spatial Tissue Optimization Slide & Reagent Kit	10× Genomics	Cat#1000193
Visium Spatial Tissue Optimization Slide & Reagent Kit	10× Genomics	Cat#1000193

**Deposited data**		

Raw immunoblotting images	This study	Mendeley Data: https://doi.org/10.17632/yd7bb3shn5.1
Paired snATAC-seq and snRNA-seq of colorectal cancer liver metastases	This study	Array Express: E-MTAB-13651, E-MTAB-13652 https://doi.org/10.17632/yd7bb3shn5.1
Spatial transcriptomics of colorectal cancer liver metastases	This study	Array Express: E-MTAB-13655
Colorectal cancer single cell RNA-seq	Che et al.,^19^ Lee et al.,^17^ Pelka et al.,^18^ Wu et al.,^20^ Sathe et al.,^30^ Wang et al.,^29^ Moorman et al.,^15^ Giguelay et al.^31^	GEO: GSE178318, GSE132465, GSE132257, GSE144735, GSE178341, GSE225857, GSE158692 dbGaP: phs001818.v3.p1 http://www.cancerdiversity.asia/scCRLM http://humantumoratlas.org/publications/hta8_crc_moorman_2024
Colorectal cancer snATAC-seq	Becker et al.^28^	GEO: GSE201349
Healthy colon scRNA-seq	Elmentaite et al.,^96^ Smillie et al.^97^	https://www.gutcellatlas.org/
Healthy colon snRNA-seq	Hickey et al.^98^	Single Cell Portal: SCP259
Colorectal cancer spatial transcriptomics (10× Visium)	Ozato et al.,^59^ Valdeolivas et al.,^63^ Fleischer et al.,^65^ Wu et al.^20^	NBDC Human Database: E-GEAD-579 GEO; GSE132465 Array Express: E-MTAB-12022, E-MTAB-12043 http://www.cancerdiversity.asia/scCRLM
Paired ATAC-seq and RNA-seq of colorectal cancer single crypts	Heide et al.^45^	Mendeley Data: https://doi.org/10.17632/7wx3chtsxx.2
Mouse high relapse cell scRNA-seq	Cañellas-Socias et al.^14^	Array Express: E-MTAB-11302
JUND ChIP-seq	Gertz et al.^106^	GEO: GSE32465
HNF4A ChIP-seq	Yan et al.^107^	GEO: GSE49402
Colorectal cancer RNA-seq	TCGA	https://www.cancer.gov/ccg/research/genome-sequencing/tcga
GRCh38-2020-A	10× Genomics	https://www.10xgenomics.com/support/software/cell-ranger/downloads/cr-ref-build-steps
Code	This study	https://github.com/EfremovaLab/CRC_metastasis

**Experimental models: Cell lines**		

CRC17_LM_PDO	This study	NA
CRC21_LM_PDO	This study	NA
T45	Gift from Hans Clevers	NA
L-WRN	ATCC	Cat#CRL-3276

**Oligonucleotides**		

RT-qPCR primers	This study	Table S10

**Recombinant DNA**		

pINDUCER20-GFP-aFOS	Gift from Andrew Sharrocks	NA

**Software and algorithms**		

Scanpy v1.9.1	Wolf et al.^82^	https://github.com/scverse/scanpy
Scrublet v0.2.3	Wolock et al.^83^	https://github.com/swolock/scrublet
scVI v0.16.4	Lopez et al.^84^	https://github.com/scverse/scvi-tools
Harmonypy v0.0.5	https://github.com/slowkow/harmonypy	https://github.com/slowkow/harmonypy
Infercnv v0.3.0	https://github.com/icbi-lab/infercnvpy	https://github.com/icbi-lab/infercnvpy
Milo v1.6.0	Dann et al.^24^	https://github.com/MarioniLab/miloR
10× Genomics Cell Ranger ARC v2.0	10× Genomics	https://www.10xgenomics.com/support/software/cell-ranger-arc/downloads
Squidpy v1.2.2	Palla et al.^100^	https://github.com/scverse/squidpy
Seurat v4.1.0	Hao et al.^88^	https://github.com/satijalab/seurat
Signac v1.5	Stuart et al.^90^	https://github.com/stuart-lab/signac
ArchR v1.0.1	Granja et al.^87^	https://github.com/GreenleafLab/ArchR
MACS2 v2.2.7.1	Zhang et al.^91^	https://pypi.org/project/MACS2/
Integrative Genomics Viewer	Robinson et al.^92^	https://igv.org/
HOMER v4.11	Heinz et al.^94^	http://homer.ucsd.edu/homer/
SCENIC+ v1.0.1	Bravo González-Blas et al.^41^	https://github.com/aertslab/scenicplus
pySCENIC	Kumar et al.^95^	https://github.com/aertslab/pySCENIC
scAR	Sheng et al.^99^	https://github.com/Novartis/scar
10× Space Ranger v.1.3	10× Genomics	https://www.10xgenomics.com/support/software/space-ranger/downloads/space-ranger-installation
cell2location v0.1	Kleshchevnikov et al.^60^	https://github.com/BayraktarLab/cell2location
SpatialDE2 v1.1.1.dev103 + g78da0ac	Kats et al.^61^	https://github.com/PMBio/SpatialDE
GSEApy v0.10.8	Fang et al.^102^	https://github.com/zqfang/GSEApy
CellPhoneDB v3.1.0; database v4.0.0	Efremova et al.^66^	https://github.com/Teichlab/cellphonedb
NicheNet v2.0.0	Browaeys et al.^67^	https://github.com/saeyslab/nichenetr
TCGAbiolinks	Colaprico et al.^104^	https://bioconductor.org/packages/release/bioc/html/TCGAbiolinks.html
DESeq2	Love et al.^105^	https://github.com/thelovelab/DESeq2
CMScaller	Eide et al.^27^	https://github.com/Lothelab/CMScaller
Trimmomatics	Bolger et al.^108^	https://github.com/usadellab/Trimmomatic
Bowtie2	Langmead et al.^109^	https://github.com/BenLangmead/bowtie2
Samtools	Li et al.^110^	https://github.com/samtools/samtools
Picard tools	https://github.com/broadinstitute/picard	https://github.com/broadinstitute/picard
Bedtools	Quinlan et al.^93^	https://github.com/arq5x/bedtools2
UCSC tools bedgraphtobigwig	https://github.com/ucscGenomeBrowser/kent	https://github.com/ucscGenomeBrowser/kent
Deeptools	Ramírez et al.^111^	https://github.com/deeptools/deepTools

### Experimental model and study participant details

#### Human tissue samples

Metastatic colorectal cancer tissue was provided by the Barts Cancer Tissue Bank (Research Ethics Committee approval, 2014/LO/2031 (City and Hampstead) and renewed 2019/LO/1700, www.cancertissuebank.org; CTB approval 2020/05/QM/ME/P/FreshTissue and 2021/01/QM/EM/P/Blood&Tissue). We accessed archived frozen tissue samples and collected fresh tissue samples from the Royal London Hospital, Barts Health NHS Trust. Fresh tissue samples were flash frozen in a dry ice/ethanol bath and stored at −80°C.

### Method details

#### Organoid establishment and culture

Organoids were established from mCRC patient tissue as described previously.^78^ Tissue was dissociated by cutting the tissue into small pieces and then adding 10 mL organoid complete medium (Advanced DMEM/F-12 [Thermo Fisher Scientific, 11540446], 2 mM L-Glutamine [Thermo Fisher Scientific, 25030081], 1% penicillin/streptomycin [Thermo Fisher Scientific, 15140122], 1 mM N-Acetly-L-Cysteine [Sigma-Aldrich, A9165], 10 mM HEPES [Thermo Fisher Scientific, 11560496], 1× B-27 [Thermo Fisher Scientific, 17504044], 1× N2 [Thermo Fisher Scientific, 17502048], 50 ng/mL EGF [Thermo Fisher Scientific, PMG8041], 10 nM Gastrin I [Sigma-Aldrich, SCP0152], 500 nM A83-01 [CliniSciences, 04–0014], SB202190 [Cambridge Bioscience, 10010399], 10 mM nicotinamide [Sigma-Aldrich, N0636], 25% L-WRN conditioned media^79^]) supplemented with 100 μg/mL collagenase IV (Thermo Fisher Scientific, 17104019) and 0.1 mg/mL primocin (InvivoGen, ant-pm-1). The tissue and digestion buffer were then transferred to a gentleMACS C tube (Miltenyi Biotec, 130-093-237) and run on the h_tumor_01 program on a gentleMACS dissociator (Miltenyi Biotec). The sample was then incubated at room temperature for 30 min with gentle shaking before running on the h_tumor_02 program, followed by a further 30 min incubation at room temperature, followed by running the h_tumor_03 program. The dissociated tumor specimen was then filtered through a 100 μm strainer and centrifuged at 800 RCF for 2 min before removing the supernatant. 1mL ACK lysis buffer was added and the sample was incubated at room temperature for 5 min to lyse red blood cells. 29 mL PBS was added and the sample was resuspended followed by centrifugation at 800 RCF for 2 min. The sample was resuspended in 5 mL PBS followed by centrifugation at 800 RCF for 2 min. Cells were resuspended in BME-2 (Biotechne, 3533-005-02) and cultured at 37°C, 5% CO_2_.

Organoids were passaged by resuspending BME-2/organoid domes in cold PBS, followed by centrifugation at 500 RCF, 5 min and the supernatant was discarded. Organoid pellet was resuspended in 1 mL TrypLE (Thermo Fisher Scientific, 12604013) and incubated for 15 min at 37°C, followed by centrifugation at 800 RCF for 5 min. Single cells were then resuspended in BME-2 at a density of 500 cells/μL BME-2 and seeded in 10–20 μL BME-2/organoid domes. Organoids were cultured in organoid complete medium.

For 2D culture, plates were coated in collagen I (Nitta Gelatin, 631-00651-FJ) by diluting collagen I gel 1:10 in 1 mM HCL according to the manufacturer’s instructions.

#### Lentiviral transduction

pINDUCER20-GFP-aFOS was packaged into lentivirus as described previously.^80^ Lentivirus was resuspended in transduction medium (complete organoid medium supplemented with 10 μM Y-27632 [MedChem Express, HY-10071] + 8 μg/mL polybrene [Merck Millipore, TR-1003-G]), and organoids were transduced based on a published protocol.^81^ Organoids were harvested and dissociated into single cells before resuspending 1 × 10^5^ cells in 250 μL lentiviral suspension, followed by centrifugation at 75 RCF at 22°C for 1 h. Organoids were then incubated at 37°C, 5% CO_2_ for 1 h, followed by centrifugation at 800 RCF for 5 min. Organoids were then resuspended in 200 μL BME-2 and incubated in complete organoid medium. 3 days later, 500 μg/mL G418 (Thermo Fisher Scientific, 11811023) was added to select transduced organoids.

To induce GFP-aFOS expression, organoids were cultured in media without L-WRN conditioned media to avoid potential doxycycline contamination from FBS. Instead of L-WRN conditioned medium, medium was supplemented with 100 ng/mL murine WNT3A (Peprotech, 315-20), 100 ng/mL murine Noggin (Peprotech, 250-38), and 500 ng/mL human R-spondin-1 (Peprotech, 120-38). aFOS-GFP was induced using 2 μg/mL doxycycline.

#### Live cell imaging and 2D culture

Organoids were dissociated into single cells and 1 x 10^5^ cells were seeded per well into a collagen I coated 12-well plate and incubated overnight. Media was changed to L-WRN free medium and cells were treated with 0 (control) or 2 μg/mL doxycycline. Cell growth was monitored using an IncuCyte S3 (Sartorius) using a 10× objective.

#### Reverse transcription quantitative PCR (RT-qPCR)

To compare 3D and 2D culture, 1 x 10^5^ single cells were cultured in 3D (200 μL BME-2) or 2D (collagen I coated 6-well plate) for 1 week. For GFP-aFOS induction in 3D culture, 1 x 10^5^ single cells were seeded in 200 μL BME-2 and cells were incubated for 6 days prior to treatment with doxycycline for 24 h in L-WRN free medium. For GFP-aFOS induction in 2D culture, 4 x 10^5^ cells were seeded into collagen I coated 6-well plates and incubated overnight prior to treatment with doxycycline for 24 h in L-WRN free medium.

RNA was extracted using a RNeasy Plus Mini Kit (Qiagen, 74136) and RT-qPCR was performed using a Luna Universal One-Step RT-qPCR Kit (NEB, E3005). 10 μL reactions were performed with 41 ng RNA and 400 nM forward and reverse primers in a 384-well plate (Table S10). Reactions were run on an QuantStudio 7 (Thermo Fisher Scientific). Relative copies were estimated from a standard curve and normalised to the geometric mean of *RPLP0* and *GAPDH* housekeeping genes.

#### Small molecule inhibitor treatments

For 2D culture conditions 1 x 10^4^ single cells were seeded into a collagen I coated 96-well plate and treated with inhibitors 24 h after seeding. For 3D culture conditions 1.5 x 10^3^ single cells were seeded in 3 μL BME into a 96-well plate and were grown into organoids for 5 days and then treated with inhibitors. Both organoids in 2D and 3D were treated with trametinib (MedChem Express, HY-10999-10mg) for 4 days. Relative cell number was then determined using a CellTiter-Glo 3D assay (Promega, G9681) according to the manufacturer’s instructions.

#### Immunoblotting

Cells were seeded in six-well plates in 2D (2.5 x 10^5^ cells per collagen-coated well) and 3D (1 x 10^5^ cells in 200μL BME) culture conditions. After 1 week, cells were treated either with DMSO or 20 nM trametinib for 2 h. Lysates were prepared in SDS-lysis buffer ((50 mM pH 7.4 Tris-HCl (Sigma-Aldrich, T2194), 2% SDS (Sigma Aldrich, 436143), 5% glycerol (Sigma Aldrich, G7757-1L), 5 mM EDTA (Thermo Fisher Scientific, 10031660), 1 mM NaF (Sigma Aldrich, S1504-100G), 10 mM β-glycerophosphate (Sigma Aldrich, G9422-50G), 1 mM PMSF (Sigma Aldrich, P7626-5G), 1 mM Na_3_VO_4_ (Thermo Scientific Chemicals, 10114740), protease inhibitor tablet (Roche, 11836170001) and phosphatase inhibitor tablet (Roche, 4906837001)) and centrifuged through a 0.2-μm multi-well filter plate (Cytiva, 5053) to remove DNA. The protein concentration of each sample was determined using a BCA assay (Thermo Fisher Scientific, A55864). The normalized lysates were then loaded onto precast 12-well bis-tris gels (Invitrogen, 15324604). Gels were transferred to nitrocellulose membranes (Bio Rad, 1620115) using a wet transfer system and subsequently blocked for 1 h in Intercept Blocking Buffer (IBB) (LI-COR, 927–60001) diluted 1:1 in TBS. Membranes were incubated overnight at 4°C in phospho-p44/42 MAPK primary antibody (1:1000) (Cell Signaling Technology, 4370S) prepared in a 1:1 mixture of TBS-T (TBS +0.1% Tween 20 (Sigma Aldrich, P1379-100ML)) and IBB. Following day, membranes were probed with the HRP-linked secondary antibody (1:5000) (Cell Signaling Technology, 7074S) then developed using chemiluminescent substrate (Themo Scientific, 34580) and visualised via BioRad ChemiDoc Imaging System. To quantify the total protein expression level of MAPK, the same membranes were incubated in re-blot solution (Millipore, 2504) for 15 min at room temperature to strip antibodies and re-probed with p44/42 MAPK primary antibody (1:1000) (Cell Signaling Technology, 9102S) following the same procedure described above.

#### Tissue dissociation and single cell multiomics

10× Genomics Multiome technology was used to generate paired snRNA- and snATAC-seq data from the same cell. Nuclei were isolated using a method based upon the salty EZ-10 V2 method (dx.doi.org/10.17504/protocols.io.buxnnxme). Frozen patient tissue was cut in a sterile dish on dry ice into a rice sized section and the remaining tissue was stored at −80°C. Frozen tissue was then transferred into 300 mL Salty-Ez10 Lysis Buffer (10 mM Tris-HCl pH 7.4, 146 mM NaCl, 1 mM CaCl_2_, 21 mM MgCl_2_, 0.03% Tween 20, 1% BSA, 10% EZ lysis buffer [Sigma, NUC101-1KT], 1 U/mL RNase inhibitor [Merck, 3335399001], 1 mM DTT, nuclease free water) in a 1.5 mL tube and the sample was homogenised by stroking 15× with a douncer (Fisher Scientific, 13236679), keeping the sample on wet ice. 700 mL Salty-Ez10 lysis buffer was added and the sample was pipette mixed using a wide-bore pipette tip. The sample was then incubated for 3 min on wet ice, and was pipette mixed with wide-bore tips twice during the incubation. The nuclei suspension was then passed through a 70 μm strainer (Fisher Scientific, 15346248) into a fresh 1.5 mL tube. Nuclei were then centrifuged at 500 RCF for 5 min at 4°C, before discarding the supernatant. Nuclei were resuspended in 500 mL WRB2 buffer (10 mM Tris-HCl pH 7.4, 10 mM NaCl, 3 mM MgCl_2_, 0.1% Tween 20, 1% BSA, 0.01% digitonin [Thermo Fisher, BN2006], 1 U/mL RNase inhibitor [Merck, 3335399001], 1 mM DTT, nuclease free water), gently pipetting using wide-bore tips. Nuclei suspension was then passed through a 40 mm strainer (Sigma, BAH136800040) into a fresh 1.5 mL tube. Nuclei were then centrifuged at 500 RCF for 5 min at 4°C before discarding the supernatant. Nuclei were then washed by re-suspending in 500 mL WRB2 buffer using wide-bore pipette tips and the centrifugation step was repeated. Nuclei were then re-suspended using a wide-bore pipette tip in 10–40 μL 1× nuclei buffer (10× Genomics, 2000153) depending on the size of the pellet. Nuclei were counted by diluting 2 μL of the nuclei suspension 10-fold with WRB2 buffer. 10 μL of diluted nuclei were then added to 10 μL dead cell stain (2% Ethidium homodimer-1 [ThermoFisher, E1169], 30% glycerol, nuclease free water) and counted on a Countess II FL Automated Cell Counter (ThermoFisher; AMQAF1000). Downstream processing was performed immediately using Chromium Next GEM Single Cell Multiome ATAC + Gene Expression Reagent kits (10× Genomics, 1000285) and Chromium Next GEM Chip J Single Cell Kit (10× Genomics, 1000234). ATAC and gene expression libraries were sequenced on an Illumina NextSeq 550 or Illumina NovaSeq 6000.

#### 10× Visium spatial transcriptomics library preparation

10 μm sections were taken from fresh frozen liver metastatic CRC samples from three patients. The tissue permeabilisation time was optimised using a Visium Spatial Tissue Optimization Slide & Reagent Kit (10× Genomics, 1000193), following the manufacturer’s instructions (Rev D). A 6 min permeabilisation time was selected.

Spatial transcriptomic libraries were created using a Visium Spatial Gene Expression Slide & Reagent Kit (10× Genomics, 1000187), following the manufacturer’s instructions (Rev E). Libraries were sequenced on an Illumina NovaSeq 6000.

### Quantification and statistical analysis

#### Analysis of primary CRC scRNA-seq datasets

Publicly available scRNA-seq data from primary CRC were combined from 4 studies.^17^^,^^18^^,^^19^^,^^20^ Raw scRNA-seq counts were analyzed using Scanpy^82^ (v1.9.1). Quality control and initial filtering was done on each dataset separately before integrating them into a single dataset. Scrublet^82^^,^^83^ (v0.2.3) was run per sample to identify potential doublets. The raw gene expression matrices were filtered using the following quality control criteria: (1) > 300 genes; (2) < 20% mitochondrial reads. Ribosomal and mitochondrial genes were discarded. The datasets were concatenated into a single gene expression matrix. The data were normalised with a scale factor of 10,000 and log1p-transformed. We extracted 2000 highly variable genes (HVGs) using the Seurat V3 method. The data were batch-corrected using scVI^84^ (v0.16.4) on raw counts and HVG, aligning all datasets and patients, and correcting for unwanted sources of variation: mitochondrial and ribosomal percentages. We used default parameters (one hidden layer with size 128 and latent size 10). A neighborhood graph (KNN) was built using the resulting 10 latent embeddings of all cells obtained from scVI to perform Leiden clustering, and UMAP visualisation.

To define major cell types, cells were clustered using the Leiden method (resolution parameter r = 0.2). Differentially expressed genes were identified for each cluster using Wilcoxon rank-sum test with Benjamini-Hochberg *p*-value correction in Scanpy. We selected differentially expressed genes with an adjusted *p*-value lower than 0.05 and a log2 fold-change higher than 1 and expression observed in a minimum of 10% of cells in a cluster. The transcriptomes were partitioned into 8 major cell types (epithelial, stromal, endothelial, T/natural killer (NK)/innate lymphoid cells (ILC), myeloid, mast, plasma and B cells) by comparing differentially expressed genes and canonical markers from the literature.

Subsequently, the same integration and clustering analysis was applied iteratively to the cells of each major cell type separately to identify and annotate cell states. For each cell, the cell cycle phase scores (G1, S, G2/M) were computed based on the expression of S and G2/M markers using *tl.score_genes_cell_cycle* using cell cycle genes identified previously.^85^ In the immune and stromal cell analysis, clusters with high numbers of doublet cells were removed by checking for expression of markers of more than one cell type. For the T/NK/ILC subpopulations, two clusters were removed as they exhibited markers from myeloid and T cells and B and T cells respectively. For the myeloid cells, three clusters showing hybrid transcriptional signatures (B/Myeloid, T/Myeloid, Epithelial/Myeloid) and a high scrublet score were discarded. For stromal cells, four clusters exhibiting doublet signatures were excluded.

Cancer cell states in pCRC were identified by subsetting and re-clustering epithelial cells. Cell cycle phase scores were calculated as described above and the top 2000 HVGs were determined for the epithelial cells. The number of observed genes, percentage of mitochondrial and ribosomal reads, and S-phase and G2-phase scores per cell were regressed from log1p-normalised counts using *pp.regress* and then scaled using *pp.scale*. PCA was then computed on the top 2000 HVGs. The python implementation of Harmony^86^ was then used to batch correct the data, using the *run_harmony* function with the patient of origin as the batch key. A neighborhood graph was then constructed (*pp.neighbors*) from the corrected principal components followed by UMAP representation (*tl.umap*). Cells were then clustered (*tl.leiden, resolution = 1.2*). Clusters that contained potential doublets were removed and the above steps were repeated to re-cluster the cells.

Primary tumor samples may contain normal colon cells. Therefore, copy number alterations were predicted from scRNA-seq data using the python implementation of inferCNV (https://github.com/icbi-lab/infercnvpy) to identify malignant cells. Normal epithelial cells in SMC, KUL^17^ and Pelka et al.^18^ datasets were used as a reference for inferCNV. Copy number alterations were inferred using bins of 100 genes, with a stepsize of 1 using the *inferCNV* function. CNV clustering was then performed using (*cnv.tl.pca*, *cnv.pp.neighbors, cnv.tl.leiden*). Normal reference cells were only present in 5/51 CNV clusters making up 23.0%, 8.8%, 4.0%, 0.16%, and 0.05% cells in each respective cluster. Epithelial cells present in 3 CNV clusters containing 23.0%, 8.8% and 4.0% reference cells were removed, resulting in 60,526 malignant cells.

Cell clustering steps (starting from re-computing cell cycle scores and re-calling HVGs) were then repeated, resulting in 17 leiden clusters (0–16). Cluster 16 was removed because it potentially contained doublets (stromal markers, *COL1A2*, *COL3A1*). Clusters 5 and 12 contained secretory cells which were subsetted and re-clustered (resolution = 0.4) into Enteroendocrine, Goblet and Tuft cells. Cluster 10 contained both HLA-high and iREC cells, and was sub-clustered (resolution = 0.2) into HLA-high cells (express HLA genes and ISGs, but lack pEMT genes) and iREC (express ISGs and pEMT genes). Cluster 0 contained both Colonocyte and Hypoxia cells, and was subsetted and re-clustered (resolution = 0.4) into Colonocyte (*SLC26A3*) and Hypoxia (lack *SLC26A3* expression) cells.

The abundance of cancer cell states was compared between mismatch repair-proficient (MMRp) and mismatch repair-deficient (MMRd) pCRC tumors using Milo^24^ (v1.6.0) in R. Milo was performed on Harmony-corrected scRNA-seq data, using default parameters except where specified. A k-nearest neighbor graph was constructed using *k* = 25 and *d* = 20, followed by defining neighbourhoods using prop = 0.1, *k* = 25 and *d* = 20 with refinement_scheme set as ‘graph’. Neighborhood testing was performed using fdr.weighting set as ‘graph-overlap’.

We scored the macrophage and monocyte subpopulations in pCRC and mCRC tumors for signatures derived from recurrent tumour-associated macrophage (TAM) and tumour-infiltrating monocyte subsets obtained from a single-cell RNA-seq analysis spanning over 15 tumor types (including CRC).^56^ In particular, we included signatures of lipid-laden TAMs, pro-angiogenic TAMs, inflammatory cytokine-enriched TAMs, interferon-primed TAMs, resident-tissue macrophages, classical monocytes, nonclassical monocytes and intermediate monocytes. We computed the gene signature scores for each transcriptome using Scanpy’s *tl.score_genes* function.

#### Analysis of single-nuclei multiome data

Single-cell Multiome data was pre-processed using the 10× Genomics Cell Ranger ARC (v2.0) pipeline. Cellranger-arc count was used to align reads to hg38 (GRCh38) and to generate barcode counts. Filtered feature barcode matrices and ATAC fragment files were used for subsequent analysis.

The quality of snATAC-seq data was assessed using ArchR (v1.0.1).^87^ Cells (i.e., barcodes) were retained if transcription start site (TSS) scores were greater than 4 and the number of unique ATAC fragments was greater than 1500 per cell. Following snATAC-seq barcode filtering, quality control of snRNA-seq was performed for the same cell barcodes using Seurat (v4.1.0).^88^ Cells with less than 300 genes and more than 10% mitochondrial DNA reads were removed. Mitochondrial and ribosomal genes were removed. We observed ambient RNA contamination in our Multiome gene expression data which we decontaminated using decontX.^89^ decontX also generates a decontamination score per nuclei and cells with a high decontamination score are potential doublets/low quality cells. We therefore removed nuclei with a decontX contamination score greater than 0.5.

Following decontamination of snRNA-seq data, decontaminated counts were loaded into an anndata object using Scanpy’s (v1.9.1)^82^ *read_mtx* function. Decontaminated counts were normalised (*pp.normalize_total* with scale factor of 10,000), log transformed (*pp.log1p*) and the top 2000 HVGs were identified (*pp.highly_variable_genes, flavor = ‘seurat_v3’, n_top_genes = 2000, batch_key = ‘Sample’*). scVI (v0.16.4)^84^ was used to batch correct snRNA-seq data by sample (*model.SCVI.setup_anndata*, *model.learn*). 10 latent variables were used for batch correction, and the number of genes and percentage of mitochondrial reads were regressed out by inclusion as model covariates. A neighborhood graph (*pp.neighbors*) was constructed from the latents and a UMAP (*tl.umap, min_dist = 0.3*) was embedded followed by clustering of cells (*tl.leiden, resolution = 0.5)*. Cell types were annotated based upon the expression of known marker genes.

To cluster all cells based upon snATAC-seq data, Signac v1.5^90^ was used. A peakset was formed by calling pseudobulk peaks on cell types annotated in snRNA-seq data. Signac functions *callPeaks(group.by = 'Cell_type')*, *keepStandardChromosomes (pruning.mode = "coarse")* and *subsetByOverlaps (ranges = blacklist_hg38_unified, invert = TRUE)* were used to call peaks and create a peakset. MACS2^91^ was used for peak calling. Reads in peaks were then quantified using the *FeatureMatrix* function. The Signac pipeline was then run: *FindTopFeatures(min.cutoff = 5)*, *RunTFIDF*, *RunSVD*, *RunUMAP*. Samples were then integrated using Signac: *SplitObject (split.by = ‘Sample’)*, *FindIntegrationAnchors(reduction = "rlsi", dims = 2:30)*, *IntegrateEmbeddings(dims.to.integrate = 1:30)*, *RunUMAP(dims = 2:30)*.

Following annotation of the major cell types, cancer cells were subsetted and analyzed, using Scanpy and scVI to integrate the samples as described above. Cell cycle scoring was performed using *tl.score_genes_cell_cycle* using cell cycle genes identified previously.^85^ The top 2000 HVGs were then re-identified and scVI batch correction was repeated, using 20 latent variables. The effect of the cell cycle was also used as a covariate in scVI. Doublets were removed by removing clusters that expressed marker genes for more than one cell type. Cancer cell states were annotated based upon the expression of marker genes, gene ontology analysis of the top genes in each cluster or by scoring cells for gene signatures (*tl.score_genes*). To score mCRC with pCRC malignant cell state signatures, signatures were obtained from the top 50 DEGs (FDR<0.01 and log_2_FC > 0.5) in pCRC states.

Following analysis and clustering of the snRNA-seq data, the snATAC-seq data was further processed using ArchR. Peaks were called in each cell state and a union peakset was created using the *addGroupCoverages*, *addReproduciblePeakSet(cutOff = 1* × *10*^*−5*^*)* and *addPeakMatrix* functions in ArchR; MACS2 was used for calling peaks.

To visualise snATAC-seq genome coverage, bigwig files were generated using *getGroupBW(tileSize = 50)* in ArchR and then visualised in the Integrative Genomics Viewer (IGV).^92^ Putative enhancer gene linkages (PE-GLs) were predicted in mCRC cancer cells based upon the correlation of gene expression with peak accessibility using *addPeak2GeneLinks(dimsToUse = 1:20, k = 100)*. The overlap of putative enhancers (pE) in PE-GLs with a set of chromHMM enhancers identified in CRC organoids^32^ was determined using bedtools *intersect.**^93^*

To infer changes in TF activity, chromVAR deviations enrichment analysis^33^ was calculated using ArchR. CIS-BP motif annotations were added to peaks using *addMotifAnnotations* and background peaks were calculated using *addBgdPeaks*. ChromVAR deviations were then calculated using *addDeviationsMatrix*.

For integrated analysis of transcription factor mRNA expression and motif enrichment analysis, decontaminated count data was loaded into an ArchR object using *addGeneExpressionMatrix*. TF mRNA expression was correlated with transcription factor binding motif accessibility using ArchR’s *correlateMatrices* function.

*De novo* motif enrichment analysis was carried out using HOMER (v4.11),^94^ *findMotifsGenome -size 200*.

To identify TF motifs enriched in topics and differentially accessible regions (DARs), a cistarget database was created using peaks in the mCRC cell state union peakset and the scenicplus public motif collection (v10nr_clust_public).^41^ DNA sequences of peaks in the union peakset were obtained using *bedtools getfasta.**^93^* The create_cistarget_motif_databases python script (https://github.com/aertslab/create_cisTarget_databases) was then used to create ranking and scores databases.

SCENIC+ (v1.0.1)^41^ was used to predict both chromatin regions (i.e., putative enhancers) and genes regulated by transcription factors in mCRC cell states. Rather than re-calling peaks with SCENIC+, peaks in the cancer cell state union peakset that was created with ArchR were used. Only cell barcodes that passed earlier QC filtering steps and genes expressed in a minimum of 60 cells were used. First a pycisTopic object was created using *create_cistopic_object_from_fragments*, using cancer cell barcodes and metadata, the blacklist peaks from ArchR hg38, and fragment files generated using 10× cellranger-arc. Topic modeling was used to identify variable open chromatin regions across cells, using *run_cgs_models*. 24 topics were selected and the model was added to the pycisTopic object using *add_LDA_model*. Putative enhancer regions for TF motif enrichment were identified from (1) regions assigned to topics and (2) differentially accessible regions. (1) Region-topic probabilities were obtained using *binarize_topics(method = ’ostu’)* and *binarize_topics(method = ’ntop’, ntop = 3000*). (2) DARs were determined using *impute_accessibility(scale_factor = 10*^*6*^*)*, *normalize_scores(scale_factor = 10*^*4*^*)*, *find_highly_variable_features*, *find_diff_features*. Only open chromatin regions on known chromosomes were retained. TF motif enrichment was then performed against a custom cistarget database (see above) using *run_pycistarget*. An SCENIC+ object was then created (*create_SCENICPLUS_object*) from the cistopic object, log1p-normalised snRNA-seq data, and TF motif enrichment results generated using pyscistarget. TF-to-gene adjacencies were calculated (*arboreto_with_multiprocessing --method grnboost2*) with pySCENIC^95^ and added to the SCENIC+ object using *load_TF2G_adj_from_file* to reduce SCENIC+ memory requirements. SCENIC+ was then run to identify TF regulons in the mCRC cell states using *run_scenicplus*(*biomart_host =* "http://sep2019.archive.ensembl.org/").

#### Analysis of primary snATAC-seq data

4 published snATAC-seq samples^28^ were analyzed using ArchR as described for multiome snATAC-seq data. Epithelial cells were identified and cells with greater than 3250 fragments and TSS >5 were retained. Doublets were removed using ArchR’s *filterDoublets* function. Samples were integrated using Harmony, and clusters were identified using a resolution of 0.4 before calling peaks in each cluster (q < 1 × 10^−5^) using macs2, to create a peak matrix. Cells were annotated to malignant cancer cell states by integrating pCRC malignant scRNA-seq annotations using ArchR’s *addGeneIntegrationMatrix* function before running chromVar to identify differential motif accessibility in malignant cell states.

#### Integrated analysis of normal colon scRNA-seq datasets

scRNA-seq data of healthy colon epithelial cells^18^^,^^96^^,^^97^ was integrated using Harmony in Scanpy as described for malignant cells in primary CRC datasets. Only cells with less than 10% mitochondrial reads and greater than 300 observed genes were retained. Covariates for the number of observed genes per cell and cell cycle effects (using the cell cycle difference score, calculated by subtracting G2/M score from S-phase score) were regressed out using scanpy’s *pp.regress* function. 30 Harmony-corrected principal components were used to construct a neighborhood graph. The resolution was set as 1.0 for leiden clustering.

We observed that expression of *LGR5* and other stem cell marker genes was low in stem cells in the healthy colon scRNA-seq. Therefore to identify a stem cell signature for comparing to our Multiome data from CRC liver metastases we identified a ‘multiome’ stem cell signature. snRNA-seq Multiome data of healthy colon epithelial epithelial cells^98^ was integrated using Harmony in Scanpy. snATAC-seq quality control metrics were calculated using ArchR and scRNA-seq quality control metrics were calculated using Scanpy. Cells were retained with a TSS score >5, number of ATAC fragments >2000 and number of observed genes >400. Ambient RNA was decontaminated using scAR.^99^ Mitochondrial and ribosomal genes were removed and samples were integrated using Harmony as described above, but using 25 principal components. Cells were clustered using the leiden method with a resolution of 0.1. Epithelial cells were then sub-clustered, using 20 principal components and a resolution of 0.4 for leiden clustering. A stem cell cluster was then identified based on the expression of marker genes (*LGR5*, *ASCL2*, *SMOC2*) and Seurat’s *FindAllMarkers* function was used to identify a stem cell signature.

#### Integration of published and multiome liver metastatic CRC data

To build a comprehensive scRNA-seq reference dataset for TME of liver metastatic CRC tumors, we integrated our snRNA-seq data with publicly available scRNA-seq datasets^19^^,^^20^ using scVI (v0.16.4). Each cell source was processed independently with Scanpy workflow (v1.9.1) before integrating them into a single batch-corrected dataset. While the counts were decontaminated for single-nuclei data, raw counts were used for single-cell data. We extracted 2000 HVGs using the Seurat V3 method. We log-normalised the raw counts using a scale factor of 10,000. The data were batch-corrected using scVI^84^ (v0.16.4) on raw counts and HVGs only, aligning all datasets and patients using cell source and patient as categorical covariates, and correcting for unwanted sources of variation: mitochondrial and ribosomal percentages. We used the default parameters (1 layer of size 128, 10 latent variables). The resulting scVI latent space of size 10 was used to build a KNN graph (n_neighbors = 15) and to perform UMAP visualisation and Leiden clustering (r = 0.2). Major cell types were annotated based on differentially expressed genes for each cluster and expression of canonical makers from the literature. Stromal, endothelial, myeloid, T/NK/ILC and hepatocytes cells were re-analysed separately, repeating batch correction with scVI, dimensionality reduction and Leiden clustering to annotate fine-grained cell states.

Malignant cells from published datasets^19^^,^^29^^,^^30^^,^^31^ of liver metastatic CRC tumors were integrated with scVI (v0.16.4) using each patient as a batch. We excluded cells from one patient who had mixed neuroendocrine adenocarcinoma (MANEC) histology, as described in the original study.^30^ We log-normalised the raw counts using a scale factor of 10,000 and extracted 2000 HVGs using the Seurat V3 method. Covariates for the number of observed genes per cell and cell cycle effects were regressed out. Mitochondrial and ribosomal genes were removed. Cells were clustered using the default louvain method with a resolution of 1. Cancer cell states were annotated based upon the expression of marker genes, gene ontology analysis of the top genes in each cluster or by scoring cells for gene signatures.

The decontaminated data from Moorman et al.^15^ of matched normal colon, primary and liver metastatic CRC tumors was processed using the original publication quality control thresholds (remove all droplets with posterior probability of containing cells ≦0.5 according to CellBender; remove droplets with <200 total counts, <200 total genes expressed or of which the libraries comprised >50% mitochondrial RNA). The data was integrated with scVI (v0.16.4) using each patient as a batch. We log-normalised the raw counts using a scale factor of 10,000 and extracted 2000 HVGs using the Seurat V3 method. Mitochondrial and ribosomal genes were removed. Cells were clustered using the leiden method with a resolution of 0.8. Malignant cells from both primary CRC and liver metastasis were then extracted and reanalysed by integration with scVI, using each patient as a batch. As in the original publication, iterative rounds of clustering and filtering to remove low-quality or apoptotic cells that were patient-specific. Cells were clustered using the leiden method with a resolution of 1. Cancer cell states were annotated based upon the expression of marker genes or by scoring cells for gene signatures.

#### Differential gene expression in sc/sn-RNA-seq datasets

Differentially expressed genes were identified in sc/sn-RNA-seq datasets using a Wilcoxon rank-sum test using Seurat’s *FindAllMarkers* (logfc.threshold >0.25, min.pct = 0.1) function.

#### Visium spatial transcriptomics analysis

Using 10× Space Ranger (v.1.3), count matrices for each sample were generated. FASTQ files were aligned to the human genome reference version GRCh38-2020-A. Automated tissue detection was performed, and the spot locations were aligned to the fiducial border spots in the H&E slide image to select spots located on the tissue. This generated data from 3 patients containing 1192 (CRC08), 937 (CRC09), and 1774 (CRC11) spots. Each Visium sample was processed independently using Scanpy (v1.9.1). Basic filtering was performed by discarding ribosomal and mitochondrial genes. We filtered out genes expressed in less than 3 spots and spots with fewer than 5 genes expressed. Segmentation was performed using the H&E image to approximate the number of nuclei per spot using Squidpy (v1.2.2) pipeline.^100^ Downloaded publicly available 10× Visium data were also processed in the aforementioned manner: four primary CRC samples capturing the tumor core and the invasive edge derived from one patient,^59^ six primary CRC samples derived from 3 patients^63^ for which spot histological annotations were available, three primary CRC samples derived from three patients,^29^ three liver metastatic CRC samples derived from three patients,^20^ and five liver metastatic CRC samples derived from five patients.^65^ In primary CRC Visium samples, normal spots were excluded.

We visualised the spatial distribution of signatures derived from 41 meta-programs obtained from a single-cell RNA-seq analysis of 24 tumor types (including CRC).^101^ We computed the gene signature scores for each spot using Scanpy’s *tl.score_genes* function.

To assign cell types and cancer cell states annotated by our scRNA-seq analysis to spots, we used the deconvolution-based method cell2location (v0.1).^60^ Leveraging our annotated scRNA-seq reference, cell2location estimates the abundance of each cell type at each spot. Briefly, cell2location estimated cell type signatures from our raw count scRNA-seq dataset, removing genes expressed in less than 5 cells. Gene expression profile at each spot was decomposed into a weighted linear combination of cell type signatures. Each Visium sample was analyzed separately. Additionally, to infer common patterns across a given tumor site and spatial neighbourhoods (either primary CRC or liver metastatic CRC), we performed a joint inference by integrating and normalising Visium data across four primary CRC samples and six liver metastatic CRC samples respectively. We used raw spatial mRNA counts, filtered to genes shared with the scRNA-seq data. Cell2location uses priors on the tissue and experiment quality, such as the number of cells per spot. We determined the average number of nuclei per spot upon nuclei segmentation analysis (*n* = 3) and set the regularisation of within-experiment variation in RNA detection sensitivity (α = 20) while the remaining hyperparameters were set to default. The model was trained for 30,000 iterations using GPU acceleration. We visualised the cell abundance and the absolute amount of mRNA, which represents the amount of mRNA contributed by each cell type in each location.

We used SpatialDE2 (v1.1.1.dev103 + g78da0ac)^61^ on raw mRNA counts to identify tissue regions in two Visium samples capturing the tumor core and the invasive edge in primary CRC (A1 and C1) and two Visium samples capturing different growth patterns in liver CRC metastasis (P13 and LM4), as this method takes into account spatial information. Briefly, the model is based on a Bayesian hidden Markov random field and leverages a graph representation of Visium data, assigning a cluster label to each spot based on its gene expression profile and its neighboring spots. Each sample was analyzed separately. We computed spatially variable genes on the slide and retained those with an adjusted *p*-value lower than 0.001. We used the top 2000 spatially variable genes to construct a graph which embeds the spatial relationships among spots. SpatialDE2 determined regions in each Visium slide with a spatial smoothness parameter s (s = 2 for A1, s = 0.5 for B1, s = 0.7 for C1 and D1 and P13, s = 0.2 for LM4). Clusters were merged to identify spatial clusters matching histological annotations.

We identified differentially expressed genes between the two spatial clusters denoting the invasive edge and the tumor core using Wilcoxon rank-sum test with Benjamini-Hochberg *p*-value correction in Scanpy. We selected differentially expressed genes with an adjusted *p*-value lower than 0.05 and a log2 fold-change higher than 0.25 and expression observed in a minimum of 10% of spots in a spatial cluster. We performed gene set enrichment analysis of the differentially expressed genes at the invasive edge and the tumor core using GSEApy (v0.10.8),^102^ and ‘MSigDB_Hallmark_2020’ and ‘KEGG_2021_Human’ gene sets.

To identify spatial cellular neighbourhoods, the subsequent analyses were applied separately to Visium data from primary CRC samples and to Visium data from liver metastatic CRC samples. Joint analysis of the Visium samples from the same tumor site enabled us to identify common patterns across samples. To identify colocalization of cell types and to build cellular neighbourhoods, we used the mRNA counts contributed by each cell state in each spot estimated by cell2location to partition each Visium slide into distinct cellular niches using SpatialDE2 (s = 0.1 for joint primary CRC analysis, s = 1.2 for joint liver metastatic CRC analysis). We built cellular neighbourhoods based on the estimated cell type abundance profiles of the spot itself and the surrounding neighbors, with the underlying assumption that spots having similar cell type abundance profiles will be grouped together. We leveraged the 5% percentile of the posterior distribution of the mRNA counts estimated by cell2location, i.e., the number of mRNA molecules contributed by each cell state in each spot.

Differences among cellular neighbourhoods in terms of cell abundance in pCRC samples capturing the tumor core and invasive edge were tested with Kruskal-Wallis test followed by *post hoc* Dunn test to quantitatively assess the spatial distribution of iRECs and the surrounding TME cells. *p* values were adjusted for multiple hypotheses with Benjamini-Hochberg procedure.

Additionally, to validate the identified cellular neighbourhoods, we used the non-negative matrix factorisation (NMF) module from cell2location to identify spatial co-occurrence of cell types. NMF decomposes cell type abundance estimates from cell2location into factors of cell types that colocalise. This model assumes an additive decomposition, entailing that multiple factors can co-exist at a single spot. The model was trained for a range of {5, …, 20} factors. We chose the decomposition into 7 factors for the joint analysis of the primary CRC analysis, and 10 factors for the joint liver metastatic CRC analysis as these configurations captured a reasonable number of microenvironments without splitting the cell states into many distinct factors.

We identified differentially expressed genes between the spatial cellular neighbourhoods using Wilcoxon rank-sum test with Benjamini-Hochberg *p*-value correction in Scanpy. We selected differentially expressed genes with an adjusted *p*-value lower than 0.05 and a log2 fold-change higher than 0.25 and with expression observed in a minimum of 10% of spots in a spatial neighborhood. We performed gene set enrichment analysis of the differentially expressed genes in each cellular neighborhood using GSEApy (v0.10.8),^102^ and ‘MSigDB_Hallmark_2020’ and ‘KEGG_2021_Human’ gene sets.

To assess the association between cancer cell state abundances and histological annotation patterns in pCRC, we leveraged a “validation cohort” - the Visum dataset^63^ containing histopathological annotations for each spot. This data contains 6 Visium samples from pCRC tumors of 3 patients. A pathologist manually categorised each spot based on the cellular morphology and tissue type using the H&E stained tissue sections. Three annotated tumor regions were considered: tumor (spots containing >90% epithelial cells), tumor & stroma_IC low (mixed spots containing <90% tumor, <90% stroma, immune cells (IC) in stroma <10%), and tumor & stroma_IC med to high (mixed spots containing <90% tumor, <90% stroma, IC in stroma 20–90%). Differences among tumor regions in terms of cancer cell state abundance were tested with Kruskal-Wallis test followed by *post hoc* Dunn test. *p* values were adjusted for multiple hypotheses with Benjamini-Hochberg.

#### Spatially resolved ligand-receptor interactions analysis

Spatial analysis of liver CRC metastases from patient samples identified cellular niches of segregated cancer cell states and cell subpopulations of the tumor microenvironment. Two different computational methods, CellPhoneDB (v3.1.0, database v4.0.0)^66^ and NicheNet (v2.0.0),^67^ were leveraged to investigate cell-cell interactions in the cellular neighborhood surrounding the iREC cancer cell state. First, to predict the potential ligand-receptor interactions between cell types of the tumor microenvironment (senders) and the iREC cancer cell state (receiver), CellPhoneDB was performed on the identified cellular neighborhood surrounding the iREC cancer cell state. The ligand-receptor interactions were inferred using our single-cell transcriptomics dataset of liver CRC metastases, by focusing on the cell types and cancer cell states that were congregating in the cellular neighborhood. Ligand-receptor interactions satisfying the following criteria were selected: (1) all ligands and receptors were expressed in at least 10% of the cells of each cell state; (2) the ligand-receptor interactions between two cell states were inferred using the statistical analysis method in CellPhoneDB with a *p*-value threshold of 0.05; (3) ligand-receptor interactions were pruned based on mean expression levels. Second, the predicted interactions were further filtered using NicheNet, retrieving ligand-receptor interactions whose downstream TF is active, as inferred by SCENIC+. Combining TF activity to ligand-receptor interactions using NicheNet highlighted the relevant interactions that activate downstream signaling in the responder cell state. Specifically, ligand-receptor pairs known to induce AP-1 regulon and NF-κB regulon expression in the iREC state were investigated. We considered genes positively regulated by AP-1 and NF-κB respectively. Inferred ligands were ranked according to the prior potential score, i.e., how well a ligand induces the expression of target genes of the AP-1 regulon and the NF-κB regulon respectively. Additionally, ligands were not only prioritised based on their potential score but also according to the ligand and receptor gene expression. We computed the average scaled gene expression values of ligands in senders and of corresponding receptors in the iREC state and other cancer cell states. A final set of relevant ligand-receptor interactions satisfying the following criteria were retrieved: (1) common ligand-receptor interactions between NicheNet and CellPhoneDB analyses that were statistically significant in CellPhoneDB analysis; (2) interactions inferred by NicheNet that were not present in the CellPhoneDB database (3) the average scaled expression of the corresponding receptor was higher in the iREC state relative to other cancer cell states. A circos plot using R package circlize (v0.4.15) was designed to highlight the main target genes for the predicted set of ligands. Ligands were assigned to senders by computing ligand specificity. The ligands that were expressed in more than one sender were labeled as common ligands.

We performed gene set enrichment analysis of the predicted ligands in the cellular neighborhood colocalising with iREC cancer cell state using GSEApy (v0.10.8), and ‘MSigDB_Hallmark_2020’ and ‘KEGG_2021_Human’ gene sets.

We computed spatial enrichment of potential ligands predicted to influence the iREC phenotype in the cellular neighborhood C_k_ containing iREC using the spatial gene expression of liver metastatic 10× Visium samples. The spatial neighborhood analysis was carried out on all liver metastatic samples together based on the cell type abundance, while spatial ligand enrichment was computed for each liver metastatic sample separately. We log-normalised the raw counts using a scale factor of 10,000. We implemented the ligand spatial enrichment of ligand *t* in the cellular neighborhood C_k_ (odds ratio) as the ratio of the odds of the ligand *t* being expressed by the odds of the other genes being expressed. The odds of ligand *t* being expressed in spots belonging to C_k_ were calculated by dividing the number of spots in C_k_ expressing ligand *t* by the number of spots that are not part of C_k_ expressing ligand *t*. For each ligand *t*, a 2-by-2 contingency table (2 regions: part of C_k_ and not part of C_k_; 2 categories: ligand *t* and other genes) was built. Significance was assessed with a Chi-square test using scipy.stats.chi2_contingency (SciPy v1.8.1)^103^ and by adjusting *p*-values for multiple testing with Benjamini-Hochberg correction method using statsmodels.stats.multitest.multipletests (statsmodels v0.13.2). A ligand *t* was considered statistically significantly enriched in neighborhood C_k_ with adjusted *p*-value lower than 0.05 and with a positive log odds ratio. We computed the 95% confidence interval for the log odds ratio.

#### Analysis of cancer cell state signatures in TCGA CRC bulk tumors and survival analysis

TCGA bulk RNA-seq count data was downloaded for colon and rectal cancers using the TCGAbiolinks R package.^104^ Count data was then normalised by variance stabilising transformation (VST) using DESeq2^105^ and VST counts were z-scored. Samples from patients with multiple samples in the dataset were removed.

Primary cancer cell state signatures were obtained using Seurat’s *FindAllMarkers* function, on ‘cell subtype’ level annotations for all cells in the primary CRC dataset (e.g., cell states/subtypes for epithelial, myeloid cells …). The top 50 differentially expressed genes ranked by log_2_ fold change were then used as a gene signature, and each tumor was scored for expression of the cancer cell state signature by calculating the mean of z-scored VST counts for genes in the signature. For ligands expressed by tumor microenvironment cells, each tumor was scored for expression of the ligands in the same manner. Scores for cancer state signatures and ligand expression values were then correlated using the Pearson method. To investigate cancer state gene signature expression in MSS/MSI subtypes, only TCGA bulk RNA-seq samples with MSS/MSI annotations were used.

To investigate differential abundance of proteins and phospho-peptides, we accessed TCGA reverse phase protein array (RPPA) data using cBioPortal and compared the top tertile of bulk TCGA tumors by expression of REC or iREC signature to the bottom tertile.

#### CMS classification

Single cell RNA-seq data was pseudobulked, by summing the counts for each gene in all cells from a sample. Pseudobulked counts were then converted into counts per million (cpm) and log1p transformed. Samples were then classified using the CMScaller R package.^27^ Only samples classified into a subtype were then used for the analysis. Bulk RNA-seq tumors were also classified using CMScaller.

#### Analysis of single gland RNA- and ATAC-seq data

We accessed RNA-seq and ATAC-seq data from single glands from primary CRC samples.^45^ We only retained glands from which paired RNA- and ATAC-seq data was available. A regenerative primary cancer cell state signature was obtained from the top 50 DEGs in the REC primary CRC cancer cell state and used to score each gland for expression of the REC signature as described above for TCGA RNA-seq data. Glands were then separated into tertiles, and the REC^*HIGH*^ and REC^*LOW*^ glands were identified from the top or bottom tertile respectively. Differential accessibility analysis was then performed using DESeq2, comparing REC^*HIGH*^ and REC^*LOW*^ glands. Differentially open peaks (log_2_ fold change >0.5, FDR <0.05) in REC^*HIGH*^ glands were then further analyzed using HOMER *de novo* motif enrichment to identify transcription factor motifs enriched within these regions.

#### Gene enrichment analysis

We performed gene enrichment analysis (GEA) on differentially expressed genes (log_2_ fold change >0.25, padj <0.01) using GSEApy (v0.10.8).^102^ HRCs Krt20^+/−^ gene signatures were obtained by DEG analysis of published mouse Smart-seq metastatic progression data (E-MTAB-11302) and resulting DEGs (log_2_ fold change >0.5, padj <0.01) were converted to human gene names using the biomart function in GSEApy.

#### ChIP-seq analysis

ChIP-seq data for JUND (GSE32465)^106^ and HNF4A (GSE49402)^107^ generated from HCT-116 and LoVo CRC cell lines respectively were downloaded from the NCBI sequence read archive. Reads were trimmed using trimmomatics (*LEADING:5 TRAILING:5 SLIDINGWINDOW:4:15 MINLEN:20*)^108^ and mapped to hg38 (GRCh38, refdata-cellranger-arc-GRCh38-2020-A-2.0.0) using bowtie2.^109^ Samtools^110^ was used to filter reads, keeping high quality (q30) reads, mapping to known chromosomes. Reads aligned to blacklist regions were removed using bedtools *intersect*, and duplicates were marked using Picard *MarkDuplicates*.

Peaks were called using MACS2 *callpeak* using input or IgG controls and the following parameters: *-q 0.01 -g hs -f AUTO -B --SPMR --call-summits*. The summit file was extended +/− 250bp using bedtools *slop*. Bedgraphs were converted into bigwig files for visualisation using UCSC tools *bedGraphToBigWig*. Deeptools^111^ *computeMatrix* and *plotHeatmap* were used to generate heatmaps showing TF ChIP-seq signal at chromatin regions identified in SCENIC+ JUND and HNF4A regulons.

## Supplemental information

Document S1. Figures S1–S14

Table S1. DEGs in malignant cell states in primary CRC, related to Figure 1

Table S2. Gene signatures used, related to Figure 1

Table S3. DEGs in cell types in primary CRC, related to Figure 1

Table S4. Clinical data for CRC LM specimens, related to Figure 2

Table S5. DEGs in malignant cell states in LM, related to Figure 2

Table S6. Homer *de novo* motif enrichment results for chromatin regions in putative enhancer gene linkage k-means clusters, related to Figure 3

Table S7. Transcription factor regulons predicted using SCENIC+, related to Figure 3

Table S8. Differential expression analysis of the tumor core and invasive edge in spatial primary CRC samples, related to Figure 4

Table S9. Differential expression analysis of spatialDE2 neighborhoods in LM, related to Figure 5

Table S10. List of RT-qPCR primers used, related to STAR Methods

Document S2. Article plus supplemental information
