# Supplementary material for: Phenotypic heterogeneity and plasticity in colorectal cancer metastasis
Source: Cell Genom. 2025 May 19;5(7):100881. doi: 10.1016/j.xgen.2025.100881 (PMC12278653; doi:10.1016/j.xgen.2025.100881)
Supplement: Document S1. Figures S1–S14 [file mmc1.pdf]

Cell Genomics, Volume *5*

Supplemental information

Phenotypic heterogeneity and plasticity in colorectal cancer metastasis

Samuel Ogden, Nasrine Metic, Ozen Leylek, Elise A. Smith, Alison M. Berner, Ann-Marie Baker, Imran Uddin, Marta Buzzetti, Marco Gerlinger, Cancer Tissue Bank, Trevor Graham, Hemant M. Kocher, and Mirjana Efremova

Supplementary Figures

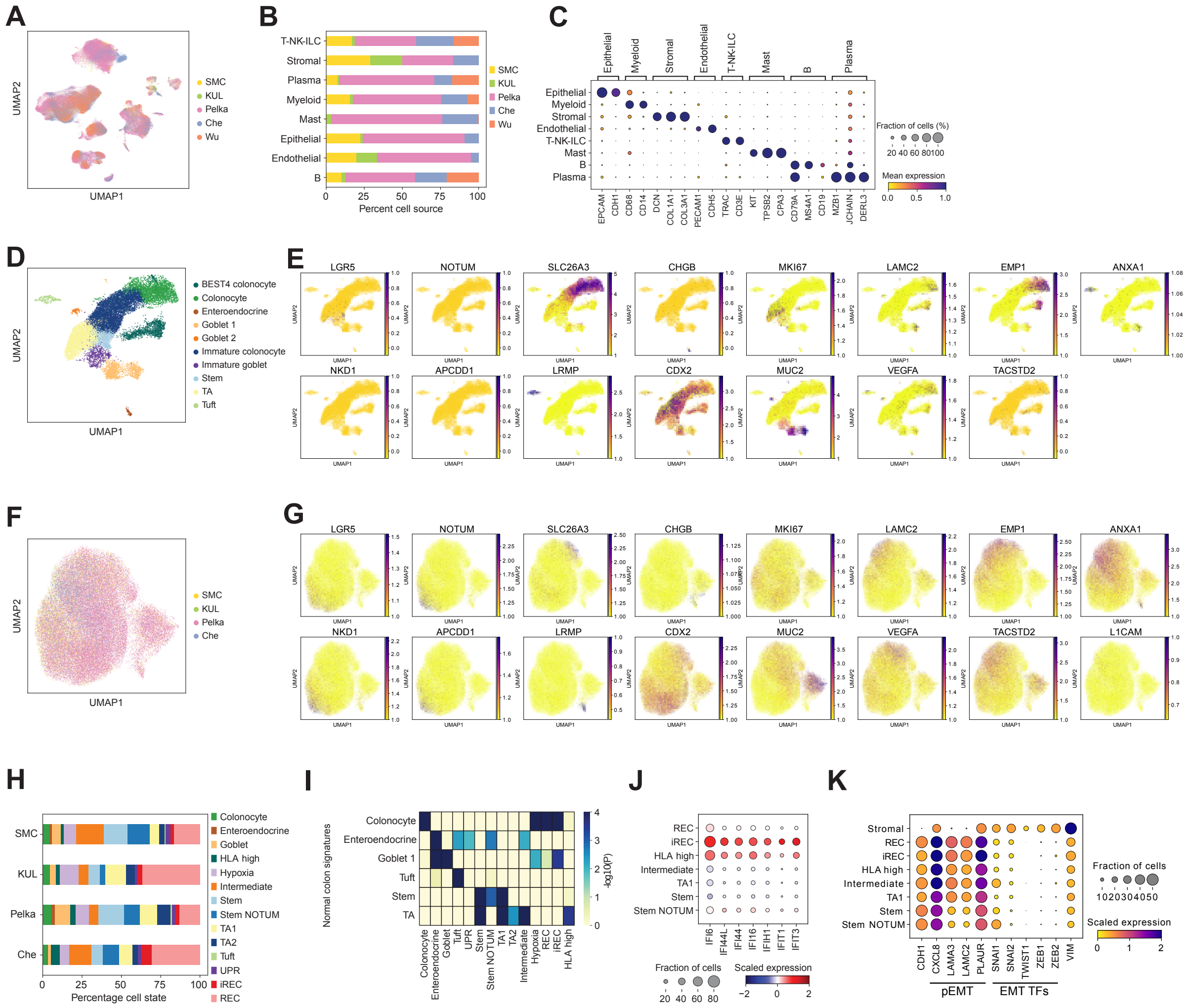

Figure S1: Cancer cell states in primary CRC (pCRC), related to Figure 1.
A. UMAP representation of integrated publicly available pCRC scRNA-seq data[17–20]. **B.** Proportions of the major cell types in pCRC tumours across the five datasets. **C.** Dotplot showing the average expression of discriminative marker genes of each major cell type (mean log1p normalised counts). The dot size indicates the fraction of cells expressing each marker in each cell type. **D.** UMAP representation of integrated publicly available normal colon epithelial cell scRNA-seq data[18,96,97]. **E.** UMAP representation of normal colon epithelial cells showing the expression (mean log1p normalised counts) of cancer cell state marker genes. **F.** UMAP representation of integrated malignant pCRC cells showing the dataset of origin. **G.** UMAP representation of malignant pCRC cells showing the expression (mean log1p normalised counts) of the indicated genes. **H.** Proportions of cancer cell states present in pCRC datasets. **I.** GEA in pCRC cell states of the expression of normal colon cell type signatures. The top 100 DEGs ranked by log2 fold change for the indicated normal colon cell type were used as signatures. **J.** Dotplot showing the scaled expression of ISGs in each of the indicated cancer cell states. **K.** Dotplot showing the expression (mean log1p normalised counts) of partial EMT (pEMT) genes, EMT transcription factors (TFs) and VIM in the indicated cancer cell states and stromal cells.

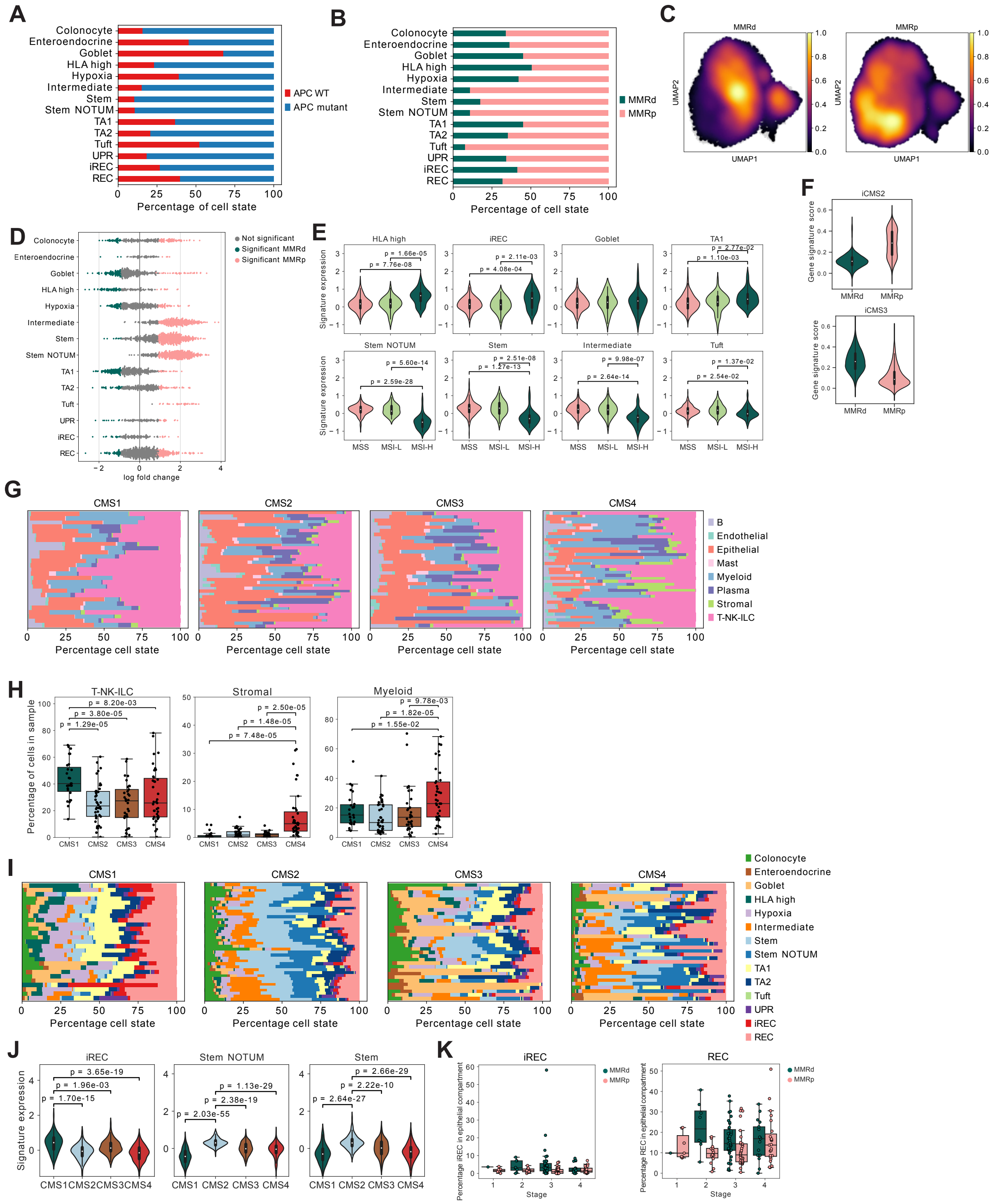

Figure S2: pCRC cancer cell states in different genomic and molecular subtypes, related to Figure 1.

A. Proportions of cancer cell states in *APC* WT or *APC* mutant tumours in SMC and KUL datasets. **B.** Proportions of mismatch repair deficient (MMRd) and mismatch repair proficient (MMRp) cells in each pCRC cell state. **C.** Density of cells in UMAP representation of MMRd (left) and MMRd (right) pCRC cells. Density is scaled between 0 and 1. **D.** Beeswarm plot showing the log fold change in abundance of MMRd and MMRp cells in pCRC cell state neighbourhoods determined using Milo[24]. Each point is a cellular neighbourhood from a KNN-graph. Neighbourhoods with a spatial false discovery rate (FDR) < 0.05 were considered as statistically significant. **E.** Expression of gene signatures for the indicated cancer cell states in TCGA bulk RNA-seq samples whether tumours are microsatellite stable (MSS, n = 185), microsatellite instability low (MSI-L, n = 43) or microsatellite instability high (MSI-H, n = 36).

Gene signatures were obtained from the top 50 DEGs ranked by log_2 fold change from analysis of pCRC malignant states and TME subpopulations (Table S3). Statistical significance was determined using an unpaired T-test. **F**. Gene signature scores for iCMS2 and iCMS3 signatures[25] in MMRd and MMRp pCRC cells. **G** and **H.** The proportions of each cell type in pCRC scRNA-seq samples classified into each CMS. Statistical significance was determined using an unpaired T-test. **I.** The proportion of cancer cell states in the epithelial compartment of pCRC scRNA-seq samples. **J.** Expression of gene signatures for the indicated cancer cell states in TCGA bulk RNA-seq samples classified into CMS. Statistical significance was determined using an unpaired T-test. **K.** The percentage of iREC and REC cells in the epithelial compartment at different tumour stages in SMC, KUL and Pelka et al. datasets.

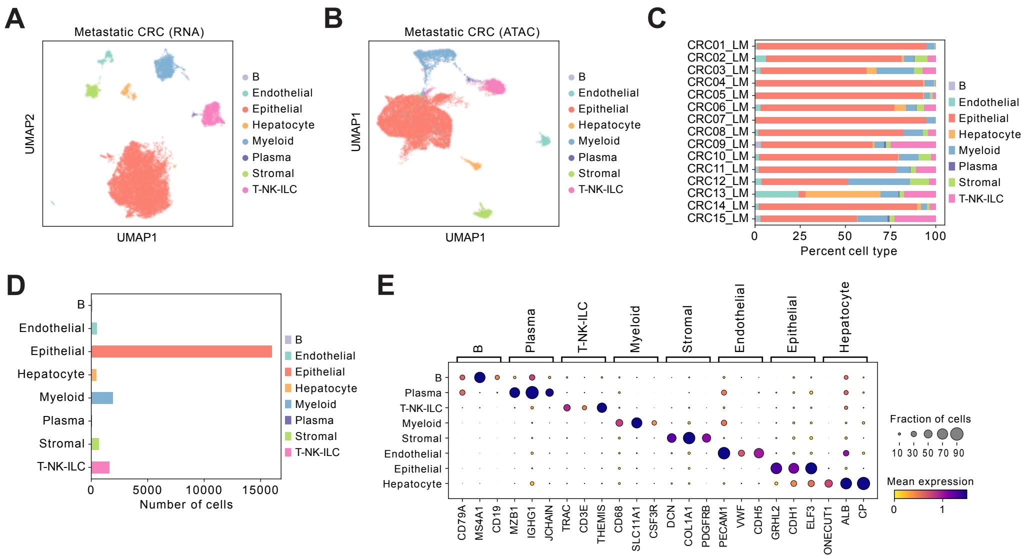

Figure S3: The single cell landscape of liver metastatic CRC in Multiome data, related to Figure 2.

A. UMAP representation of cells in the mCRC Multiome data, showing the sample of origin. UMAP is based upon the RNA modality. **B.** UMAP representation of cell types in mCRC Multiome data. The representation is based upon the ATAC modality. **C.** Proportions of cell types in the mCRC Multiome dataset across patient samples. **D.** The number of cells of each cell type in mCRC Multiome data. **E.** Dotplot showing the expression of marker genes in metastatic CRC. Mean log1p normalised counts for each cell type is shown.

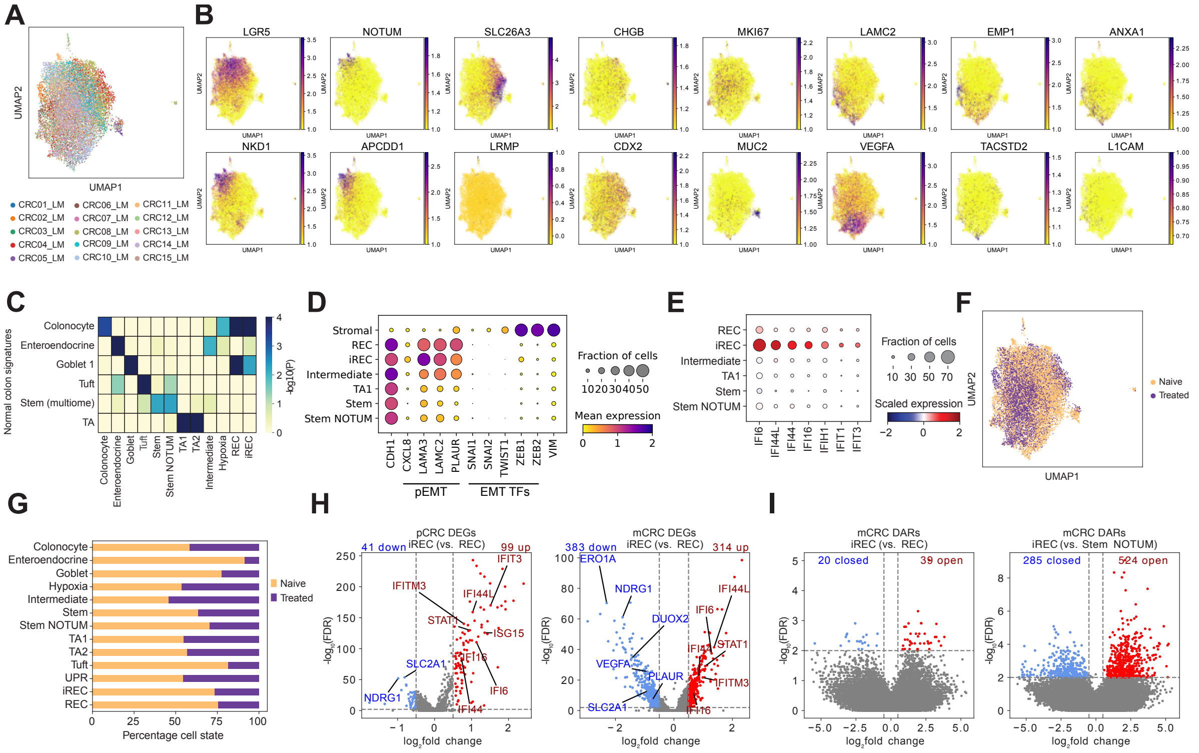

Figure S4: Cancer cell states in liver metastatic CRC, related to Figure 2.
A. UMAP representation of cancer cells in the mCRC Multiome data, showing the sample of origin. UMAP is based upon RNA modality. **B.** UMAP representation of cancer cells in mCRC, showing the expression (log1p normalised counts) of the indicated marker genes. **C.** GEA of normal colon cell type signature in mCRC cell states. **D.** Dotplot showing the expression (mean log1p normalised counts) of *CDH1*, partial EMT (pEMT) genes, EMT transcription factors (TFs) and *VIM* in the indicated mCRC cell states and stromal cells. **E.** Dotplot showing the scaled expression of ISGs in each of the indicated mCRC cell states. **F.** UMAP representation of mCRC cells showing whether patients had received chemotherapy prior to specimen collection. UMAP is based upon RNA modality. **G.** Proportions of chemotherapy-naive or treated cells in mCRC cell states. **H.** Volcano plots of DEGs between iREC and REC cancer cells in pCRC and mCRC. **I.** Volcano plots of differentially accessible regions (DARs) between iREC and REC/Stem NOTUM cancer cells in mCRC.

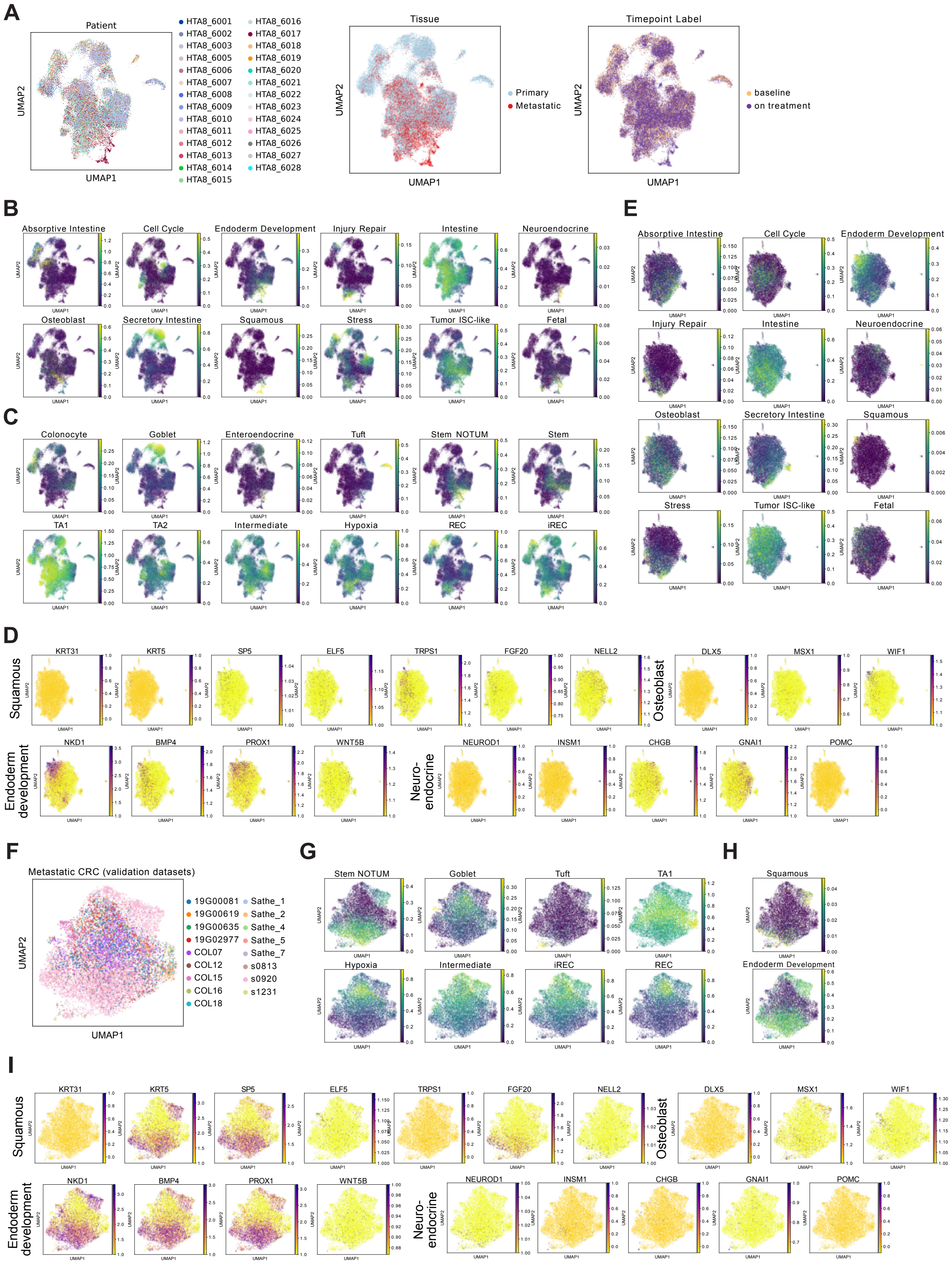

Figure S5: Expression of cancer cell state signatures in validation datasets, related to Figure 2. A. UMAP representation showing the patient, whether samples were primary or metastatic and the sample treatment status of epithelial cells in pCRC and mCRC samples in scRNA-seq dataset [15]. **B.** UMAP representation showing Hotpsot and fetal signature[15] scores in Moorman et al. dataset. **C.** UMAP representation showing multiome mCRC cancer cell state signature scores in Moorman et al. dataset. **D.** Expression of marker genes for the indicated gene modules from Moorman et al. in multiome mCRC data. **E**. Hotspot and fetal signature[15] scores in Multiome mCRC cancer cells. **F.** UMAP representation

showing the patient of origin of malignant cells in an analysis of published mCRC scRNA-seq datasets[19,29–31]. **G.** Multiome mCRC cancer cell state signature scores in published mCRC scRNA-seq datasets. Signatures were obtained from the top 50 DEGs (FDR < 0.01 and log2 fold change > 0.5) in mCRC cancer cell states. **H**. UMAP representation showing Hotspot [15] scores of the squamous and endoderm signatures in an analysis of published mCRC scRNA-seq datasets[19,29–31] . **I.** Expression of marker genes for the indicated gene modules from Moorman et al. in published mCRC scRNA-seq datasets.

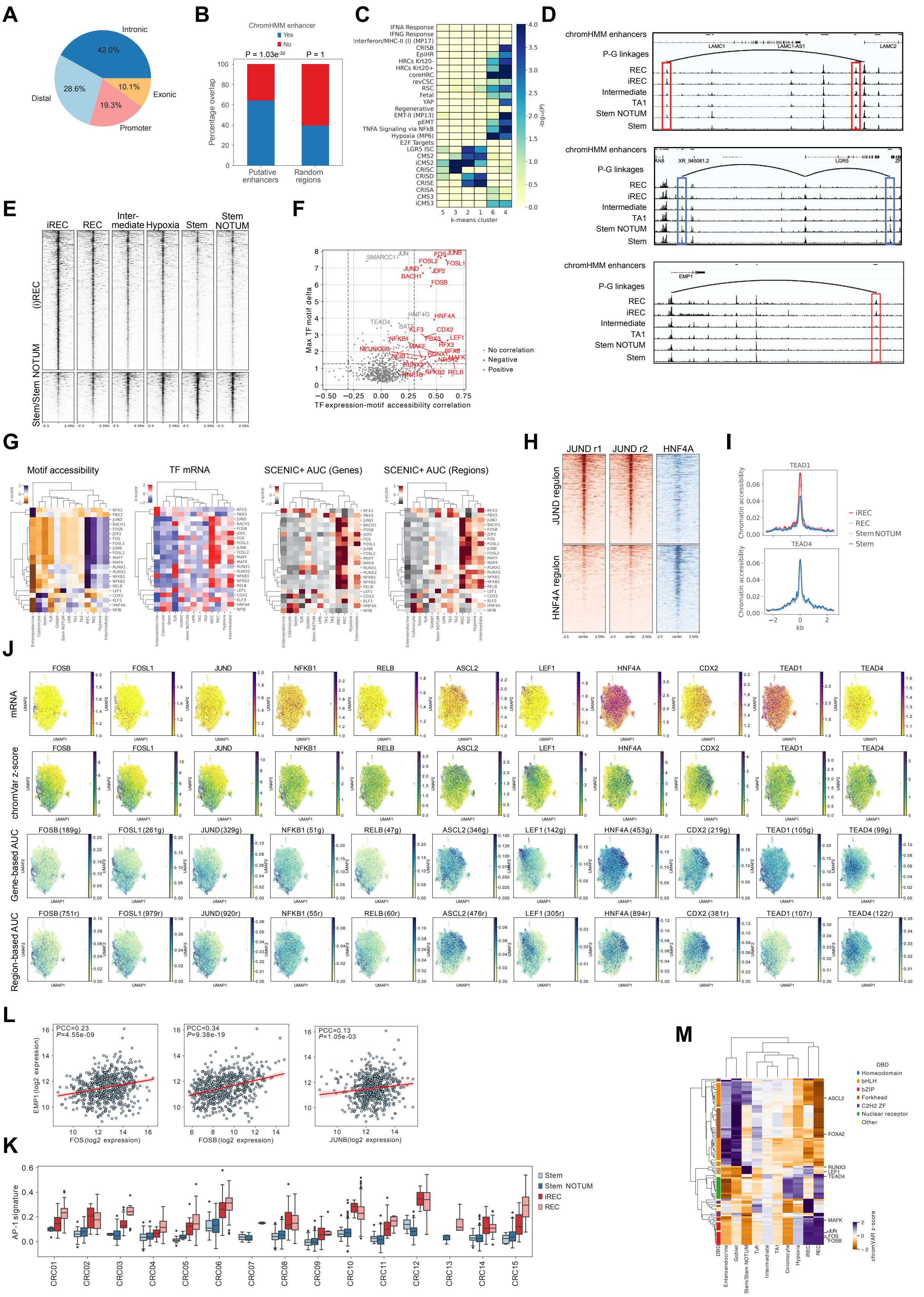

Figure S6: Transcription factors regulating cancer cell states, related to Figure 3.
A. Genomic distribution of open chromatin regions (n = 82,491) in the CRC cell state union peakset. **B.** Percentage overlap of chromatin regions in P-G linkages (n = 1,444) or a random set of chromatin regions (n = 1444) with chromHMM enhancers[32] (n = 33130). **C.** GEA of genes in k means clusters for

the indicated gene signatures. See Table S2 for the gene signatures. **D.** Chromatin accessibility at *LAMC2*, *LGR5* and *EMP1* loci in the indicated mCRC cell states. PE-GLs are shown and chromHMM enhancers[32]. **E.** Heatmap showing the chromatin accessibility of regions differentially accessible between (i)REC cells and Stem/Stem NOTUM cell states. **F.** Plot showing the maximum difference in chromVAR motif deviation z-score between CRC cell states, against correlation of chromVAR motif deviation and corresponding TF expression. **G.** Heatmaps showing (from left-right) chromVAR motif deviation z-scores, z-scored mRNA expression, enhancer driven regulons (eRegulons) z-scored SCENIC+ area under curve (AUC) scores for genes in regulons, eRegulons z-scored SCENIC+ AUC scores for chromatin regions in regulons of TFs highlighted in red in Figure S6F. **H.** JUND and HNF4A ChIP-seq signal at chromatin regions in JUND[106] or HNF4A[107] eRegulons. r1 - replicate 1, r2 - replicate 2. **I.** Accessibility of chromatin regions in the indicated SCENIC+ regulons. **J.** UMAP representation showing TF mRNA expression, and corresponding chromVAR deviation z-scores, gene-based eRegulon AUC scores, region-based eRegulon AUC scores. **K.** Expression of AP-1 experimentally determined target genes [44] in the indicated cancer cell states in mCRC Multiome data. **L.** Scatter plot showing the expression levels of the indicated AP-1 family members and *EMP1* in bulk pCRC RNA-seq (TCGA) data (n = 609). PCC – Pearson correlation coefficient. **M.** chromVAR motif deviation z-scores for the indicated pCRC cell states in pCRC snATAC-seq data[28].

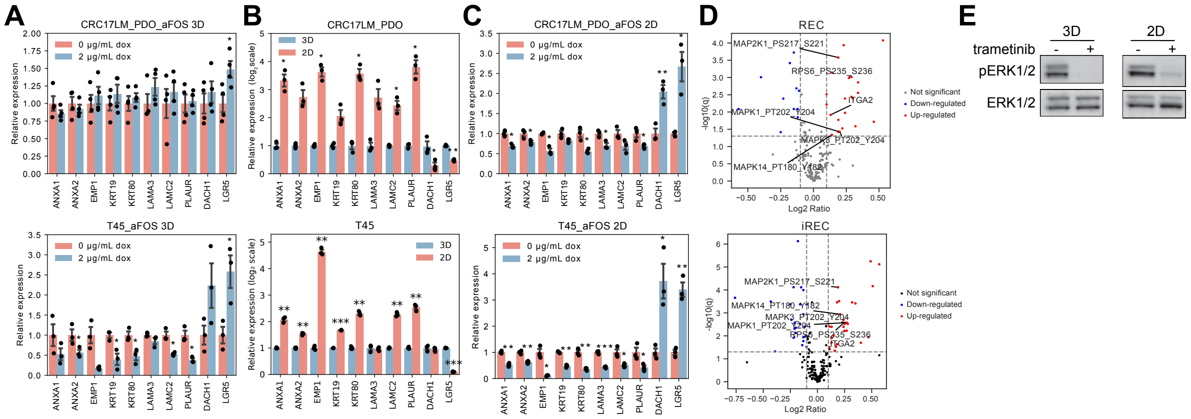

Figure S7: Inhibition of AP-1 in patient-derived organoid models, related to Figure 3.

A. RT-qPCR analysis of the indicated genes following GFP-aFOS induction by 2 µg/mL doxycycline (dox) treatment in standard organoid culture conditions (3D) in both primary (T45_aFOS) and liver metastatic organoids (CRC17LM_PDO_aFOS). Statistical significance was determined using a paired T-test, * $P < 0.05$; T45 $n = 3$; CRC17LM_PDO $n = 4$. Data are represented as mean ± SEM. **B.** RT-qPCR analysis of the indicated genes in parental T45 and CRC17LM_PDO in standard organoid culture conditions (3D) or on collagen I coated plates (2D). Statistical significance was determined using a paired T-test, * $P < 0.05$, ** $P < 0.01$, *** $P < 0.001$; $n = 3$. Data are represented as mean ± SEM. **C.** RT-qPCR analysis of the indicated genes following GFP-aFOS induction in 2D culture conditions in T45_aFOS and CRC17LM_PDO_aFOS. Statistical significance was determined using a paired T-test, * $P < 0.05$, ** $P < 0.01$, *** $P < 0.001$; $n = 5$. Data are represented as mean ± SEM. **D.** Reverse Phase Protein Array (RPPA) data showing differential abundance of phospho-peptides/proteins in TCGA bulk CRC tumours. Tumours with high expression of iREC (left) or REC (right) signatures were compared to tumours with low expression of the respective gene signature. A positive log2 ratio indicates the phospho-peptide/protein had higher abundance in tumours with higher expression of the signatures. Statistical significance was determined using a Student's T test with Benjamini-Hochberg correction. **E.** Western blot of CRC21LM_PDO treated with trametinib for 2 hours.

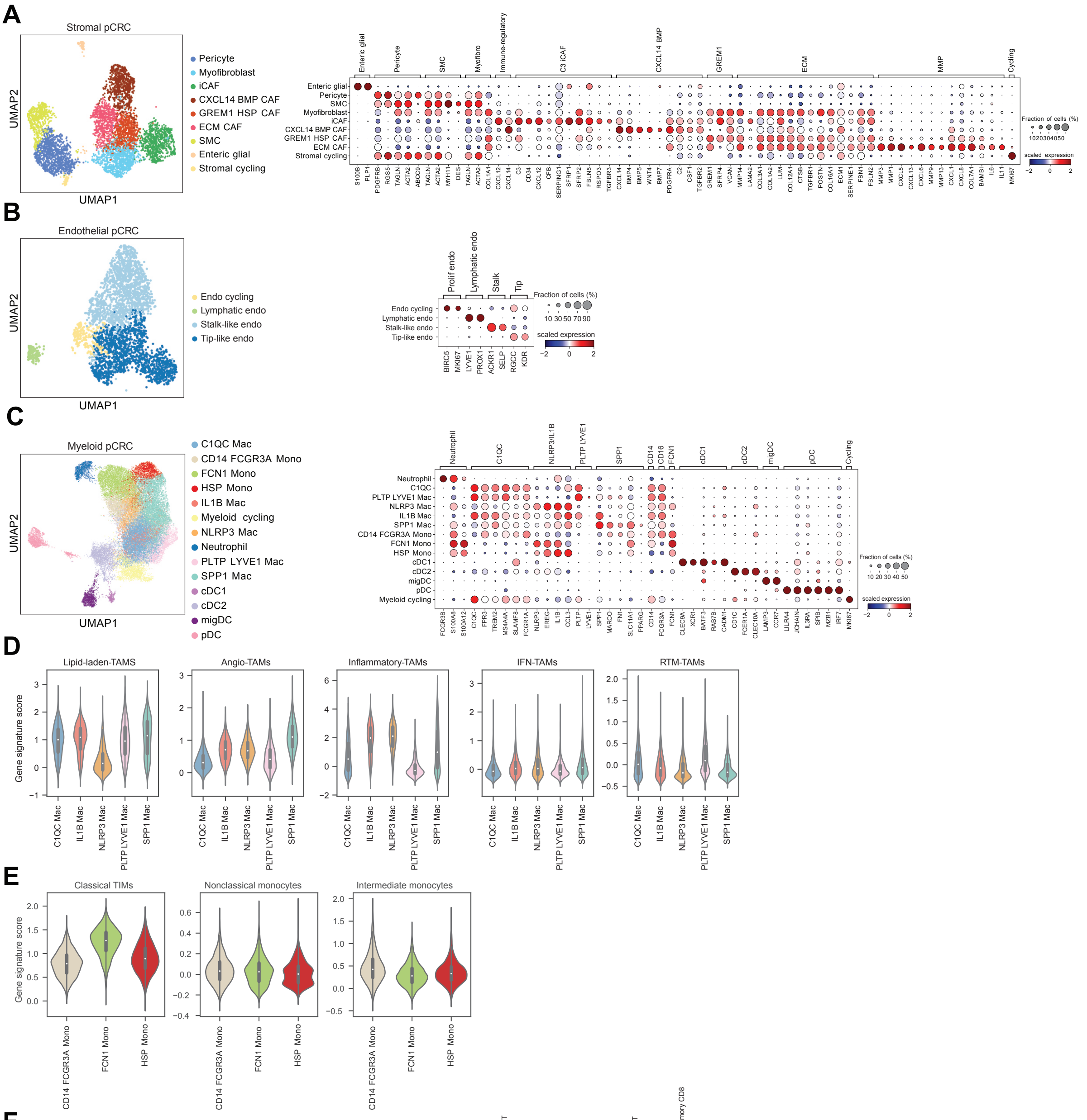

Figure S8. Characterisation of the tumour microenvironment in primary CRC (pCRC), related to Figure 4.

A. UMAP representation of stromal subpopulations from integrated analysis of publicly available datasets[17–20] (left). Dotplot showing the scaled expression of marker genes of fine-grained stromal subpopulations (right). **B.** UMAP representation of endothelial subpopulations (left). Dotplot showing the scaled expression of marker genes of fine-grained endothelial subpopulations (right). **C.** UMAP representation of myeloid subpopulations (left). Dotplot showing the scaled expression of marker genes of fine-grained myeloid subpopulations (right). **D.** Violin plots depicting gene signature scores derived from signatures of recurrent tumour-associated macrophage (TAM) subsets obtained from a single-cell RNA-seq analysis spanning over 15 tumour types (including CRC)[56] in the identified TAM subpopulations. In particular, we included signatures of lipid-laden TAMs (Lipid-laden-TAMs), pro-

angiogenic TAMs (Angio-TAMs), inflammatory cytokine-enriched TAMs (Inflammatory TAMs), interferon-primed TAMs (IFN-TAMs), resident-tissue macrophages (RTM-TAMs). **E.** Violin plots depicting scores for gene signatures of tumour-infiltrating monocyte subsets obtained from a single-cell RNA-seq analysis spanning over 15 tumour types (including CRC)[56] in the identified monocyte subpopulations. **F.** UMAP representation of T-NK-ILC subpopulations (left). Dotplot showing the scaled expression of marker genes of fine-grained T-NK-ILC subpopulations (right). Tfh: follicular helper T cell, Th: helper T cell, Tn: naive T cell, Tem: effector memory T cell, Tex: exhausted T cell, NK: natural killer cell, Treg: regulatory T cell, gdT: gamma delta T cell, ILC: innate lymphoid cell, Mac: macrophage, Mono: monocyte, DC: dendritic cell, CAF: cancer-associated fibroblast, SMC: smooth muscle cell, Endo: endothelial.

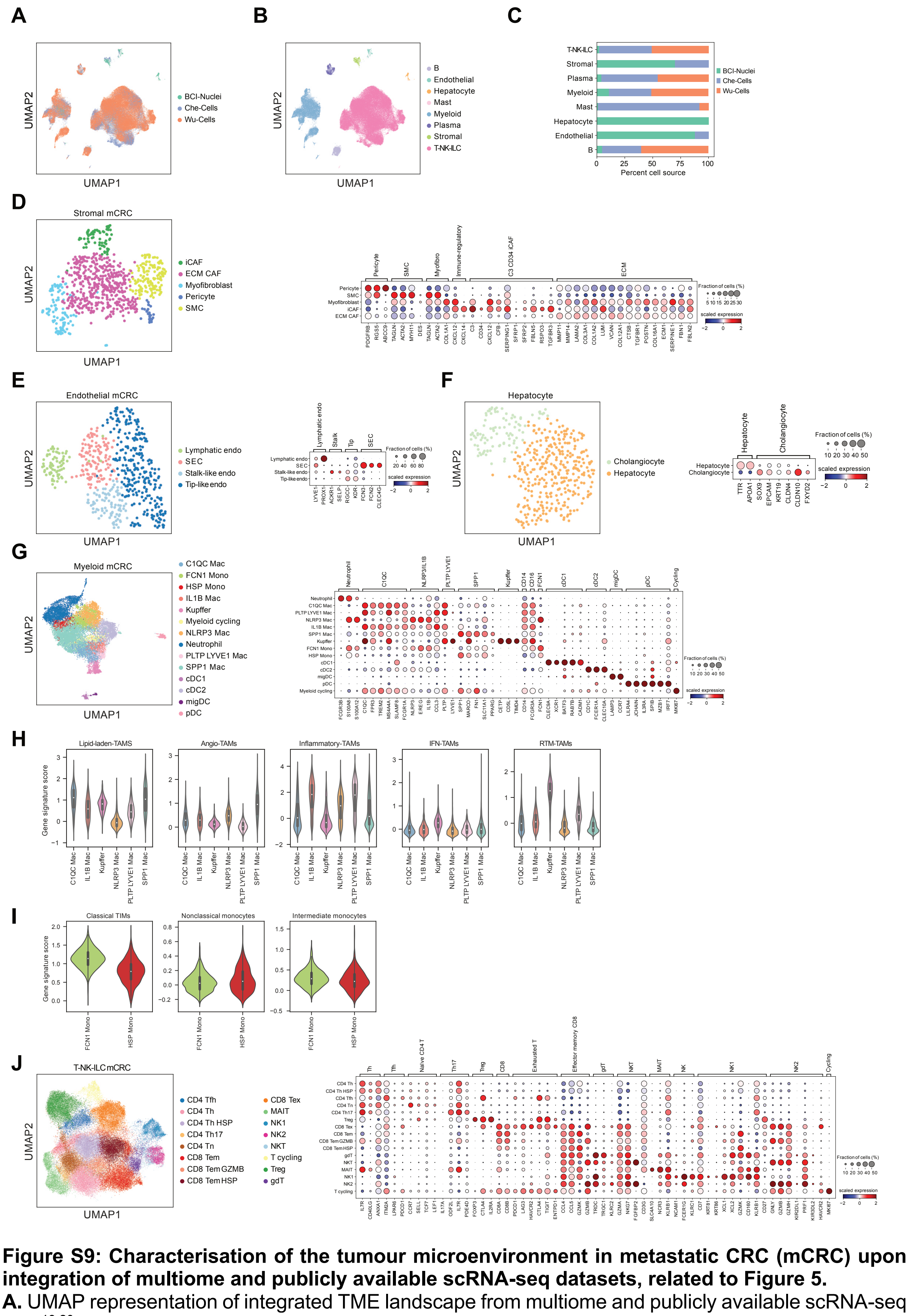

Figure S9: Characterisation of the tumour microenvironment in metastatic CRC (mCRC) upon integration of multiome and publicly available scRNA-seq datasets, related to Figure 5.
A. UMAP representation of integrated TME landscape from multiome and publicly available scRNA-seq data[19,20] of liver mCRC tumours. **B.** UMAP representation of major cell types in the TME. **C.** Proportions of the major cell types in pCRC tumours across the three cell sources. **D.** UMAP representation of stromal subpopulations (right). Dotplot showing the scaled expression of marker genes of fine-grained

stromal subpopulations (left). **E.** UMAP representation of endothelial subpopulations (left). Dotplot showing the scaled expression of marker genes of fine-grained endothelial subpopulations (right). SEC: sinusoidal endothelial cells. **F.** UMAP representation of hepatocytes and cholangiocytes (left). Dotplot showing the scaled expression of marker genes of hepatocytes and cholangiocytes (right). **G.** UMAP representation of myeloid subpopulations (left). Dotplot showing the scaled expression of marker genes of fine-grained myeloid subpopulations (right). **H.** Violin plots depicting gene signature scores derived from signatures of recurrent tumour-associated macrophages (TAM) subsets obtained from a single-cell RNA-seq analysis spanning over 15 tumour types (including CRC) in the identified TAM subpopulations[56]. Signatures of lipid-laden TAMs (Lipid-laden-TAMs), pro-angiogenic TAMs (Angio-TAMs), inflammatory cytokine-enriched TAMs (Inflammatory TAMs), interferon-primed TAMs (IFN-TAMs), resident-tissue macrophages (RTM-TAMs) are shown. **I.** Violin plots depicting scores for gene signatures of tumour-infiltrating monocyte subsets obtained from a single-cell RNA-seq analysis spanning over 15 tumour types (including CRC)[56] in the identified monocyte subpopulations. **J.** UMAP representation of T-NK-ILC subpopulations (left). Dotplot showing the scaled expression of marker genes of fine-grained T-NK-ILC subpopulations (right).

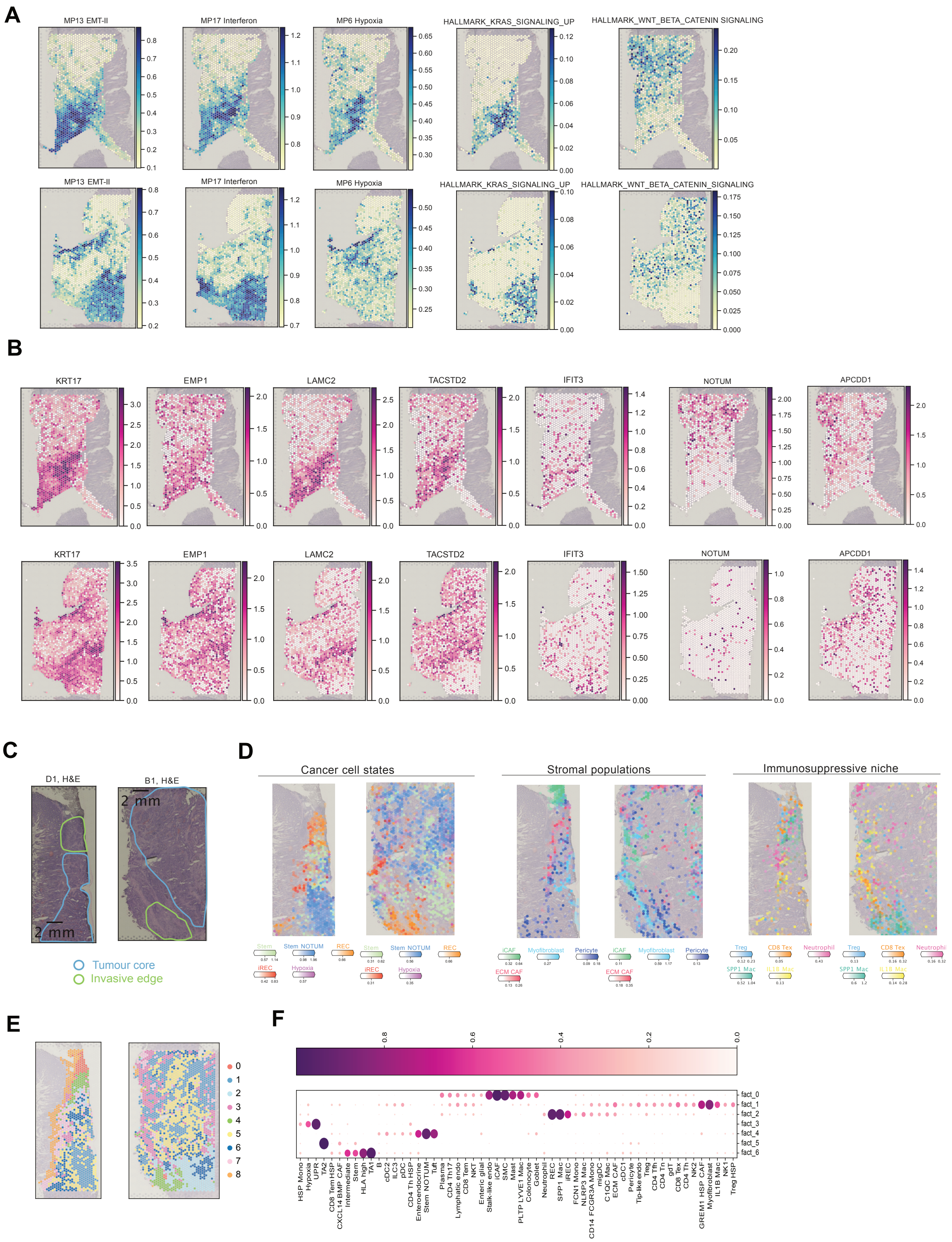

Figure S10: Spatial mapping of cancer cell states and identification of spatial neighbourhoods in primary CRC capturing the tumour core and the invasive edge, related to Figure 4.

A. Gene expression signature scores of spots for five different signatures on two Visum samples (samples A1 and C1[59]). EMT (MP13 EMT-II), interferon response (MP17 Interferon) and hypoxia (MP6 Hypoxia) cancer cell signatures are derived from a single-cell RNA-seq analysis of 24 tumour types (including CRC)[101]. Signatures for WNT signalling and KRAS signalling are obtained from MSigDB

Hallmarks. **B.** Gene expression (log1p normalised counts) of cancer state-specific marker genes associated with REC and iREC and Stem NOTUM cell states in representative sample A1 and C1. **C.** H&E staining of primary colorectal cancer samples D1 and B1 and pathologist annotations[59]. **D.** Cell abundance estimates of cancer cell states, and stromal and immune subpopulations colocalising with iRECs across spatial locations of two primary CRC samples D1 and B1 capturing the tumour core and invasive edge. **E.** Spatial cellular neighbourhood identification, shown in samples D1 and B1[59]. **F.** Cellular neighbourhood identification using the NMF module from cell2location. Dotplot depicts the relative factor loadings of each cell state, normalised across factors for each cell state. Factors correspond to cellular neighbourhoods. Factor loadings are depicted by colour and dot size.

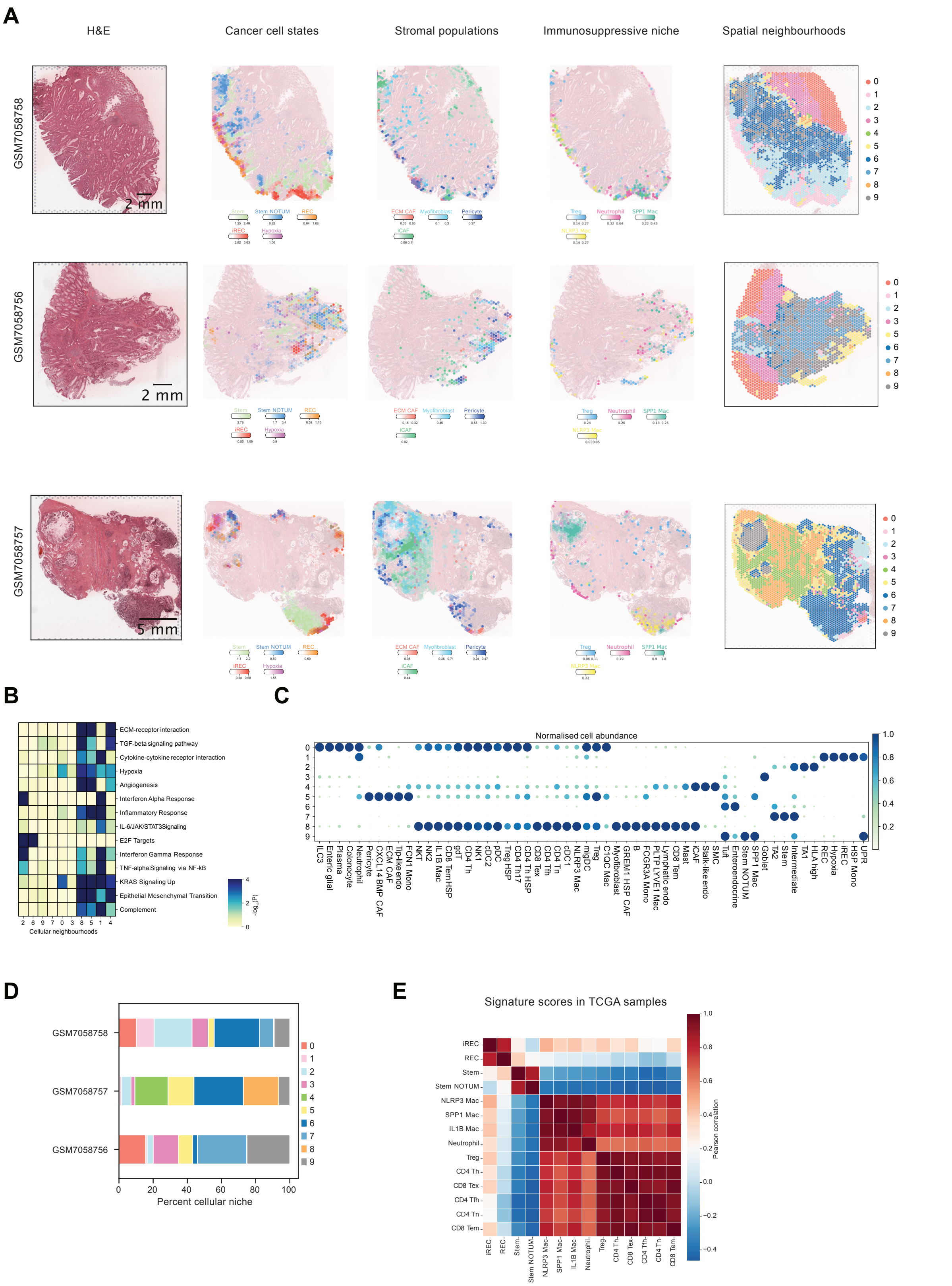

Figure S11: Identification of spatial neighbourhoods in pCRC tumours using validation dataset, related to Figure 4.
A. H&E staining (first column), cell abundance estimates of cancer cell states (second column), stromal (third column), immune populations (fourth column) and spatial neighbourhoods across spatial locations of 3 pCRC samples[29]. **B.** GEA of upregulated genes in the spatial cellular neighbourhoods. **C.** Dotplot representing the average cell abundance (dot size and colour) for each cell state, per

neighbourhood, and normalised between 0 and 1 per cell state. **D.** Proportions of the spatial cellular neighbourhoods across the 3 Visium samples. **E.** Correlation values (Pearson's coefficient) between cancer cell state signatures and immune cell signatures in TCGA bulk RNA-seq data from 609 patients.

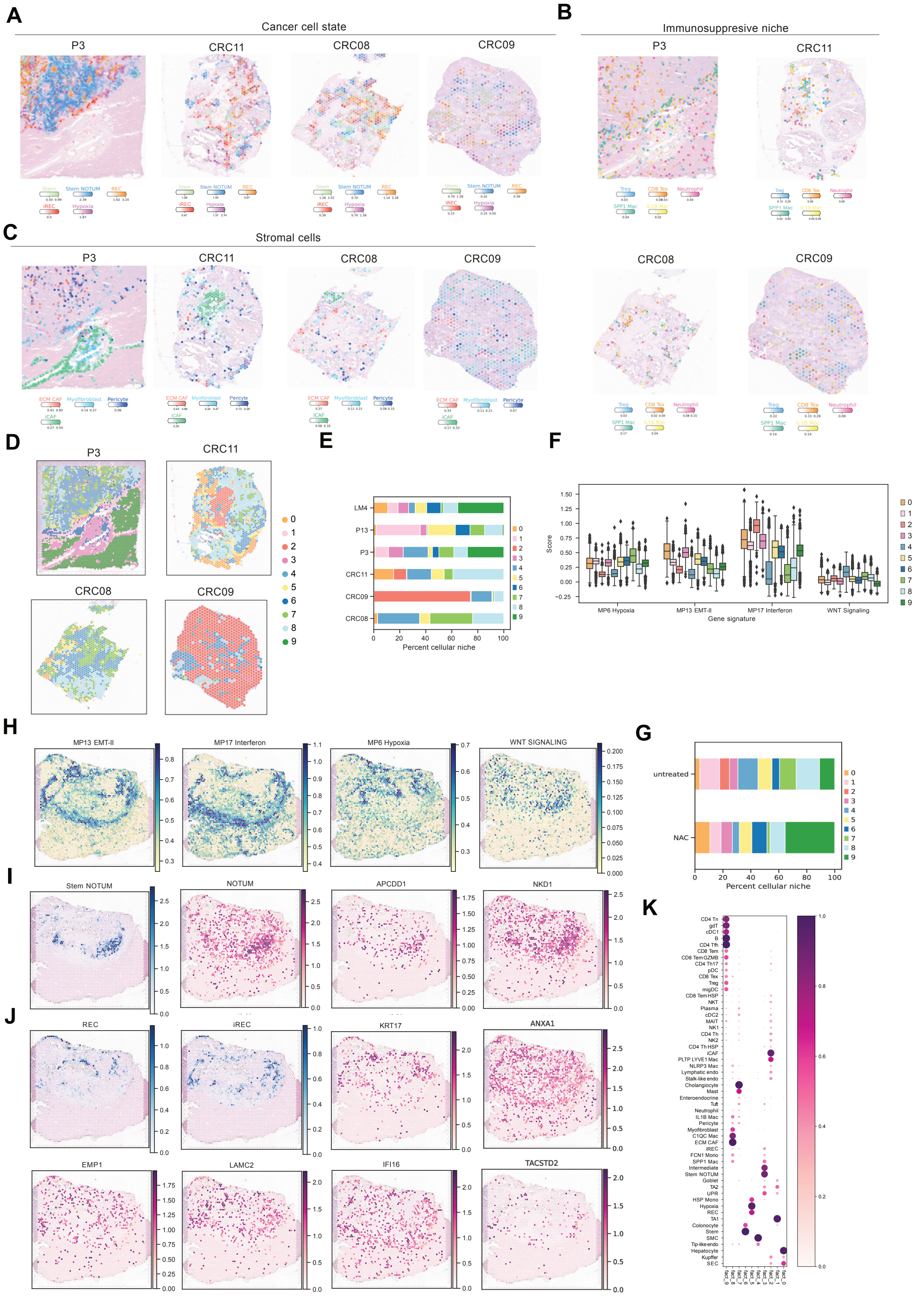

Figure S12: Spatial mapping of cancer cell states and identification of spatial cellular niches across liver metastatic CRC samples, related to Figure 5.

A. Estimated cell type abundances for distinct cancer cell states across spatial locations of samples P3[20] (untreated), CRC11 (untreated), CRC08 (untreated) and CRC09 (untreated). **B.** Estimated cell

type abundances for distinct immunosuppressice subsets across spatial locations of samples P3[20] (untreated), CRC11 (untreated), CRC08 (untreated) and CRC09 (untreated). **C.** Estimated cell type abundances for distinct stromal subpopulations (left) and immune subpopulations (right) in sample P3. **D.** Spatial cellular neighbourhoods in samples P3 (untreated), CRC11 (untreated), CRC08 (untreated) and CRC09 (untreated). **E.** Proportions of the spatial cellular neighbourhoods across the six Visium samples. **F.** Boxplots depicting four gene expression signature scores in spatial cellular neighbourhoods. The boxplots show three quartiles (lower, median, upper) of the data distribution, with whiskers extending to data points that lie within 1.5 inter-quartile ranges of the lower and upper quartile. EMT (MP13 EMT-II), interferon response (MP17 Interferon) and hypoxia (MP6 Hypoxia) cancer cell signatures are derived from a single-cell RNA-seq analysis of 24 tumour types (including CRC)[101]. Signature for WNT signalling is obtained from MSigDB Hallmarks. **G.** Proportions of the spatial cellular neighbourhoods across the six Visium samples, stratified by treatment status: untreated versus neoadjuvant chemotherapy (NAC). **H.** Gene expression signature scores of spots in representative sample LM4 for four different gene signatures[101]: epithelial-to-mesenchymal transition (MP13 EMT-II), interferon response (MP17 Interferon), hypoxia (MP6 Hypoxia) and Hallmark WNT Beta Catenin Signalling (WNT Signaling). **I.** Estimated cell type abundance and gene expression (log1p normalised counts) of Stem NOTUM-specific marker genes in representative sample LM4. **J.** Estimated cell type abundances and gene expression (log1p normalised counts) of cancer state-specific marker genes associated with REC and iREC states in representative sample LM4. **K.** Cellular neighbourhood identification using the NMF module from cell2location. Dotplot depicts the relative factor loadings of each cell state, normalised across factors for each cell state. Factors correspond to cellular neighbourhoods. Factor loadings are depicted by colour and dot size.

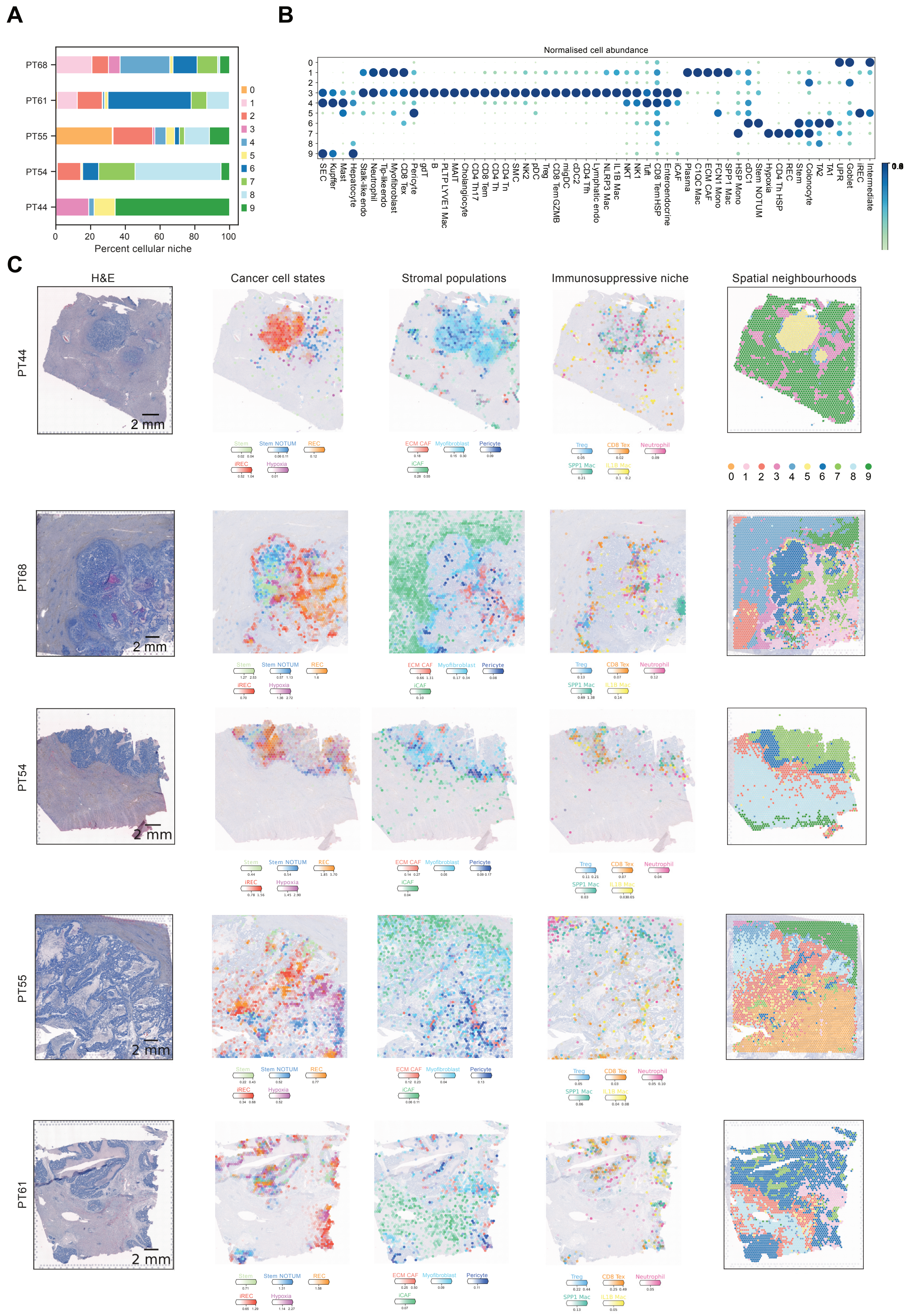

Figure S13: Identification of spatial cellular niches in validation dataset of 5 10X Visium mCRC samples, related to Figure 5.

A. Proportions of the spatial cellular neighbourhoods for each of the 5 liver mCRC samples[65]. Untreated samples: PT44, PT55 and PT61. Neoadjuvant chemotherapy (NAC) samples: PT54 and PT68. **B.** Cell type abundance across spatial cellular niches. Dotplot representing the average cell abundance (dot size and colour) for each cell state, per neighbourhood, and normalised between 0 and 1 per cell state.

C. H&E staining (first column), cell abundance estimates of cancer cell states (second column), stromal (third column), immune populations (fourth column) and spatial neighbourhoods across the 5 liver mCRC samples.

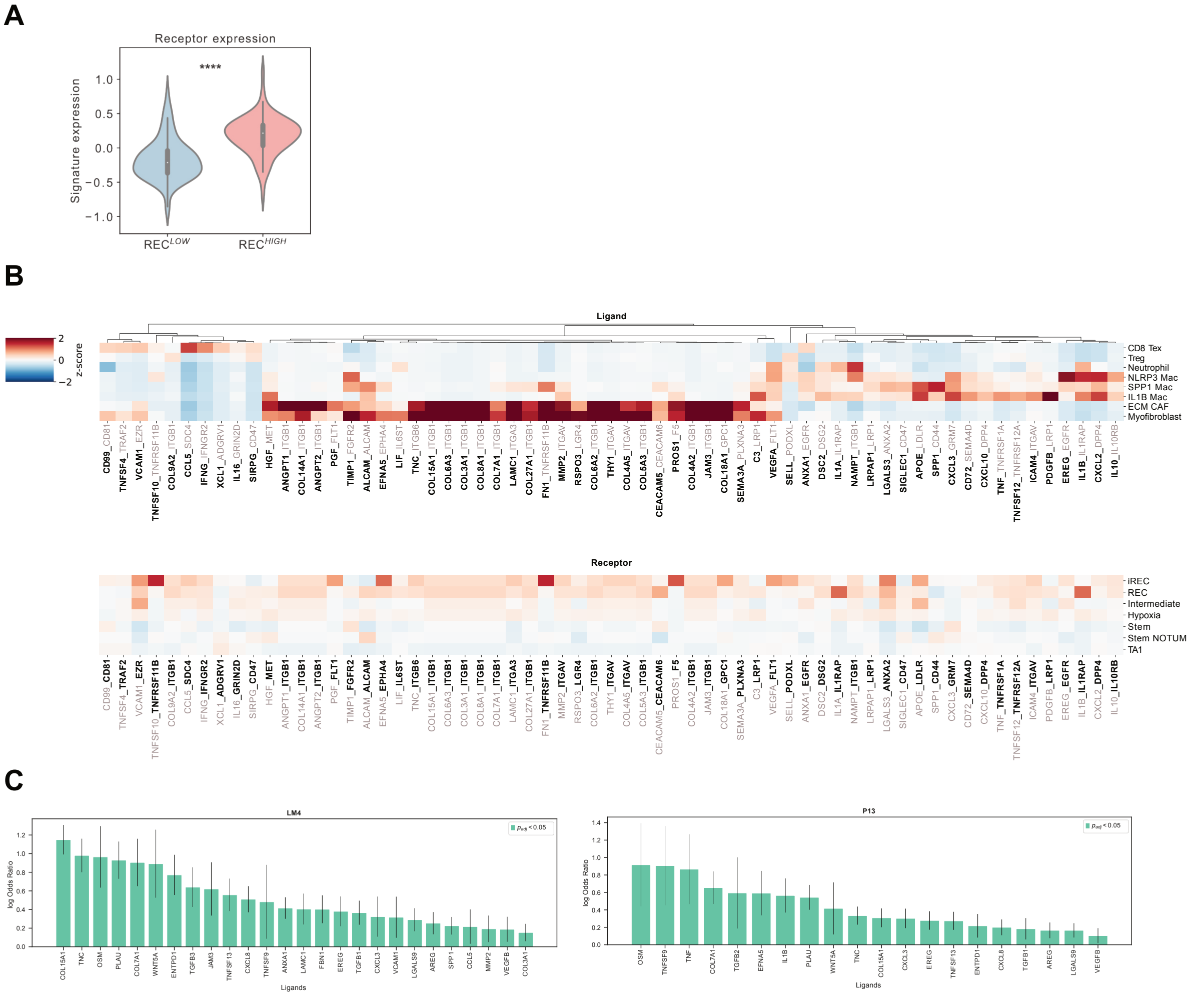

Figure S14: Spatially resolved cell-cell interactions in the cellular neighbourhood surrounding interferon response REC cancer cell state, related to Figure 6.

A. The expression of receptors shown in Figure 6a in *REC*^{HIGH} and *REC*^{LOW} single glands. Statistical significance was determined using an unpaired T-test, **** $P < 0.0001$. **B.** Heatmaps summarising the inferred spatial cell-cell interactions mediated by stromal, myeloid, CD8 exhausted T (CD8 Tex) and regulatory T cells (Treg) in the cellular neighbourhood containing iRECs, with iRECs as the receiver, using CellPhoneDB and NicheNet. Specifically, we identified potential upstream ligand-receptor pairs which can induce the NF-κB regulon program in the neighbouring pro-metastatic phenotype. Z-score of the gene expression of selected potential ligands in each cell type of the cellular neighbourhood (top panel) and z-score of gene expression of corresponding receptors in cancer cell states (bottom panel). In both heatmaps, the x-axis denotes ligand-receptor interactions, with the ligand in bold and receptor in grey for sender cells (top panel) and the ligand in grey and the receptor in bold for cancer cell states (bottom panel). **C.** Ligand spatial enrichment in the cellular neighbourhood surrounding iREC cancer state in two representative samples (LM4 and P13). Ligands predicted to induce AP-1 regulon activation and statistically significant enrichments are shown. Barplot showing statistically significant enrichments, i.e. positive log odds ratio associated with adjusted p-value < 0.05. Statistical significance is assessed with Chi-square test using a significance level of 5%. The p-values are adjusted for multiple testing using the Benjamin-Hochberg correction method. The 95% confidence interval for the log odds ratio is plotted.
